# Timber trade in 17th-century Europe: different wood sources for artworks of Flemish painters

**DOI:** 10.1038/s41598-024-68641-y

**Published:** 2024-08-06

**Authors:** Andrea Seim, Johannes Edvardsson, Aoife Daly, Pascale Fraiture, Ian Tyers, Willy Tegel, Rūtilė Pukienė, Tomasz Wazny, Maite Jover de Celis, Joost Vander Auwera, Justin Davies

**Affiliations:** 1 Chair of Forest Growth and Dendroecology, Institute of Forest Sciences, University of Freiburg, Freiburg, Germany; 2 Department of Botany, University of Innsbruck, Innsbruck, Austria; 3 The Laboratory for Wood Anatomy and Dendrochronology, Department of Geology, Lund University, Lund, Sweden; 4 International Dendrochronology Research Laboratory (Dendro.Dk), Copenhagen, Denmark; 5 Laboratory of Dendrochronology, Royal Institute for Cultural Heritage (KIK-IRPA), Brussels, Belgium; 6 Dendrochronological Consultancy, Nottinghamshire, UK; 7 Amt für Archäologie, Kanton Thurgau, Frauenfeld, Switzerland; 8 Nature Research Centre, Vilnius, Lithuania; 9 Nicolaus Copernicus University, Toruń, Poland; 10 Museo del Prado, Madrid, Spain; 11 Royal Museums of Fine Arts, Brussels, Belgium; 12 University of Amsterdam, Amsterdam, The Netherlands; 13 Department of History of Art, University of Cambridge, Cambridge, UK

**Keywords:** Art history, Dendroprovenance, Panel makers, *Quercus* spp., Jacques Jordaens, Anthony Van Dyck, Palaeoecology, Environmental social sciences

## Abstract

The former Spanish Netherlands experienced a period of social, cultural and economic prosperity in the seventeenth century, with Antwerp as its most important commercial and artistic centre. The era’s vibrant art scene, once pivotal culturally, economically, and diplomatically, now offers invaluable insights for scientific studies on art, trade, and craftsmanship. In a study on 294 panel paintings by or related to two famous Flemish artists, Jacques Jordaens (1593–1678) and Anthony van Dyck (1599–1641), we applied classical art historical techniques, archival research, dendrochronology, and the study of panel maker’s and guild marks on the painting’s reverse to gain insights into the precise time of tree felling, the geographical provenance of the wood, and the panel makers patronised by the painters. The majority of the paintings (~ 80%), which were subjected to a dendrochronological analysis, could be dated and the results accorded well with the concomitant art historical assessment on authorship. Besides an active and well-known Baltic timber trade which provided over 71% of all the planks examined, straight-grained oak trees were also sourced from western Central Europe (20%). Interestingly, planks from the Baltic and the Ardennes region (France/Belgium) were used together in three different paintings, likely cut apart from larger panels. Employing a multidisciplinary approach to a comprehensive painting collection by individual painters provides not only a new tool to determine a painting’s date and authorship but also allows for a better understanding of the contemporary timber trade and associated craftsmanship.

## Introduction

**Figure 1:**
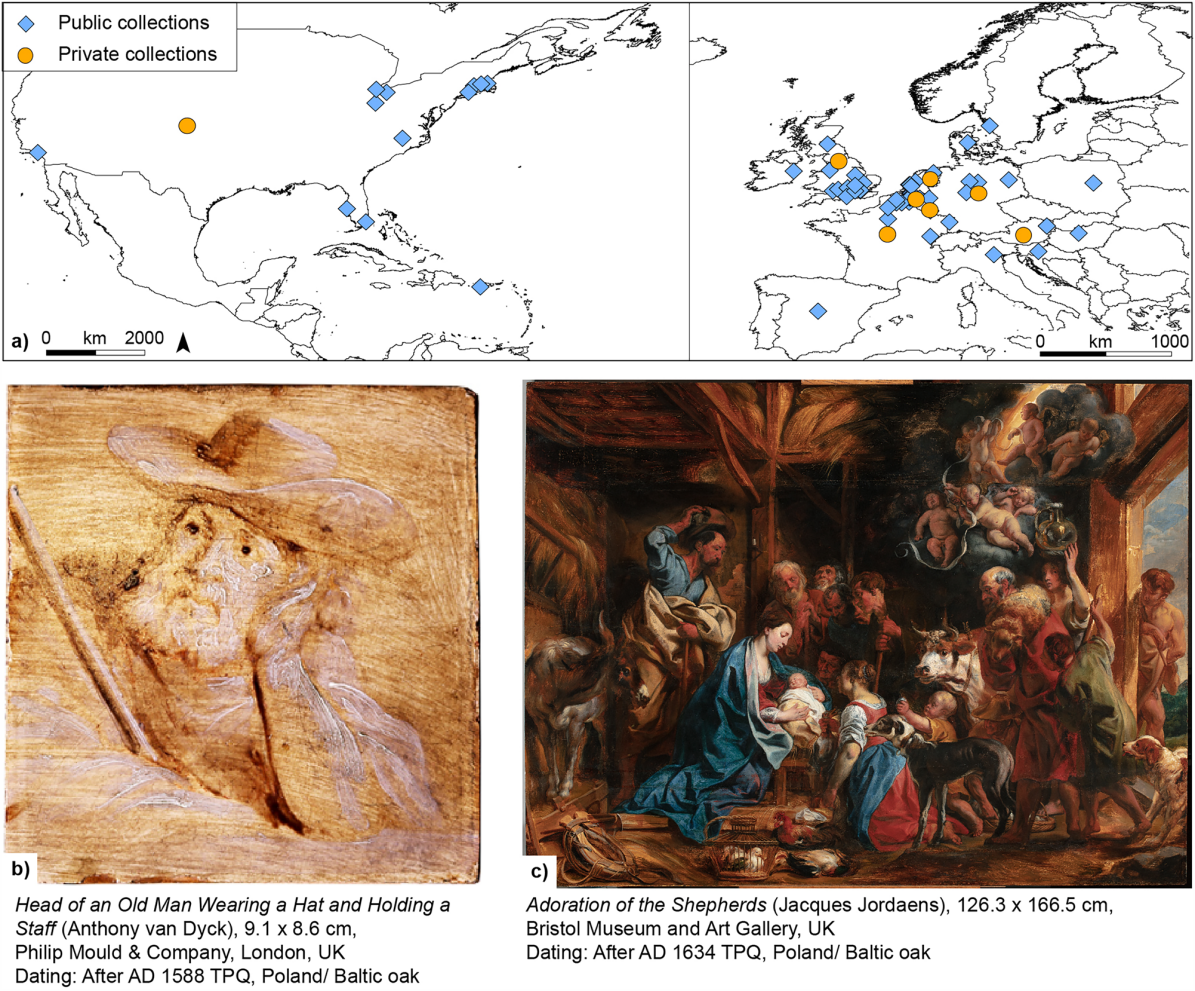
(**a**) Locations of the paintings investigated in public and private collections (see legend; private collections are given one random coordinate). The map was generated using ArcMap 10.6^12^. (**b**) Smallest and (**c**) largest panel painting investigated and dated (TPQ is *terminus post quem* (earliest possible felling date)).

During the seventeenth century and despite the Eighty Years’ War (c. 1568–1648), the Low Countries, closely corresponding to the modern-day Netherlands and Belgium, experienced a prosperous period in commerce, science, and art. The conclusion of the Twelve Years Truce between Spain and the Dutch Republic (1609–1621) further facilitated economic recovery, pausing hostilities and fostering growth. Luxury goods like the arts, based on a high degree of specialisation and a more efficient production process yielded relatively good profit margins and paintings became popular export goods. This in turn provided an advantageous context for great Flemish Baroque masters such as Peter Paul Rubens (1577–1640), Jacques Jordaens (1593–1678) and Anthony van Dyck (1599–1641). In numerous workshops that were established in thriving cities and artistic centres such as Antwerp, painters produced abundant artworks. The militant catholic church of the Counter-Reformation intensified the patronage of new altarpieces to redecorate the many churches that had fallen victim to two waves of iconoclasm, one virulent (1566), the other silent (1583). While an increasingly wealthy merchant class admired still lifes, landscape paintings, and genre scenes, royal and noble patronage favoured historical subjects and portraits^1^ (Fig. 1). The favoured support for easel paintings in the sixteenth and early seventeenth centuries was oak panels but canvas as support became prevalent as the latter century progressed. An expression of the preference of painters for panels can be found in a letter which Rubens sent to Sir Dudley Carleton on 26 May 1618 regarding his *The Expulsion of Hagar* (71 × 102 cm): ‘It is done on a panel because small things are more successful on wood than on canvas; and being so small in size, it will be easy to transport’^2^. For panels, oak (*Quercus* spp.) was the preferred tree species for painters in northern France, the Low Countries, western Germany, and England up to the end of the seventeenth century^3,4^. Panel makers and painters preferred slow-grown and old oak trees since their narrow and regular tree rings and the straightness of the grain ensured a high dimensional stability of the wooden support thus preventing deformation of the planks^3^. Moreover, such wood had a more regular and smoother surface than canvas, therefore being better for rendering minute details. Even more importantly, the pigments of the paint layers were better protected from discolouring and the preparation layer from becoming grey instead of white, because the wood blocked the oxygen in the atmosphere from intrusion from behind while the front was protected by varnish. Since oak was also the favoured tree species for construction timber, a large­scale exploitation of old oak forests started in parts of western and central Europe by the tenth century resulting in a shortage of large quantities of locally available straight­grained and knot-free construction timber, as was reported for Flanders, Belgium^5,6^. This led to the exploitation of forests in other regions of Europe and thus, a large­scale trade of construction timber developed. The timber trade in straight-grained oak trees from Poland and the other states on the Baltic Sea, here generally referred to as the Poland/Baltic region, started in the mid-fourteenth century^7–9^. An active timber trade between the Poland/Baltic states and the Low Countries and England was well established by the Kingdom of Sweden and the Polish–Lithuanian Commonwealth during the fourteenth to eighteenth centuries^10^. Since the timber was transported by sea, the felled oaks were split or sawn into planks or boards at the forest site or collection point, transported to the nearest port, and loaded onto ships^11^. Oak planks shipped from the port cities of formerly Königsberg (now Kaliningrad (Russia) and Gdansk (Poland)) measured about 280 cm (ten-foot), so-called Königsberg’s ten-foot wood (‘Coninbergh tienvoethout’)^9^.

Panel makers purchased a bundle of rough planks and further prepared them into evenly thick planks, mainly by sawing. The thickness of planks for panels of up to medium size (50 × 60 cm) varied over time and was found to be ca. 2 cm for the fifteenth century, 1 cm for the early sixteenth century and 0.4 to 0.5 cm for the seventeenth century^13^. In the seventeenth century, sawing became more common than splitting, and more half-quartered planks were produced in addition to the preferred quartered planks^13^. For larger panels, several planks were joined together relying on different joining methods, glues, and reinforcements^14^. Because panels were extremely costly, up to eight times more than canvasses of the same dimensions, concerns were raised about their quality and potential for abuse among the panel makers in the artists' and associated crafts Guild of Saint Luke in Antwerp. This prompted them to petition the authorities for a law regulating the quality and size of panels available to painters. The petition was submitted on 13th November 1617 and signed by 22 panel makers, mirror case makers and softwood frame makers^15^. It resulted in an ordinance of 11th December 1617 issued to both the Guild of Saint Luke and the Joiner’s Guild^16^. Agreed standard sizes ranged from small (*kleine stooters* of *twee en een half stuyver*: two and a half penny format, c. 40 × 31 cm), medium (*ses stuyvers maet*: six penny format, c. 60–64 × 48 cm) to large (*daeldersmaet*: one and a half guilder of thirty stuyvers, i.e. thirty penny format, c.123 × 93 cm) panels^17^. Importantly, from both a dendrochronological and art historical perspective, it decreed that the panel makers were bound to submit their joined panels (i.e. two planks or more) for inspection by the Dean of the same trade within their Guild to ensure that they did not include the softer sapwood of the tree, nor mould nor red or white worms. It was found, however, that these quality rules were not always respected or well executed (e.g. Refs.^18,19^). The Dean could break any sub-standard panels. Panels which were approved by the Dean were to be branded by him (known as the Guild mark) and, at risk of financial penalty for not doing so, the maker had to place his distinctive individual mark on the frames or panels (known as a panel maker’s mark).

As well as painting artworks themselves, Flemish masters also employed assistants in their studios to make copies. Copies were also made by painters without the instruction or supervision of the original artist. Here, dendrochronology proved to be a valuable tool for discerning later copies from original artworks by matching the tree-ring width (TRW) pattern of unknown ages (i.e. undated samples) to samples with known ages for the same geographical region^20^. This cross-dating determines the date of tree felling, which can be cross-referenced to the creative period of an artist or a panel maker and thus, provides proof as to whether a panel was created during or after a painter or panel maker’s lifetime. Dendrochronology was first applied to panel paintings in Europe in the 1960s and 1970s on German medieval painters^21^ and in the mid-1970s on fifteenth- to seventeenth-century English and Flemish artists^22^. Since then, it has become a standard method in art history for dating the wooden support of a painting.

We present the first systematic and multidisciplinary investigation at this scale conducted on any individual artists, in this case, the works on panel by and related to Jacques Jordaens (1593–1678), Anthony van Dyck (1599–1641), and their studios, as well as some collaborative works with Peter Paul Rubens (1577–1640) and copies produced by lesser-known painters such as Remigius van Leemput (1607–1675) and Theodore Russell (1614–1689). The methods employed in the research incorporated the humanities, social and natural sciences and included traditional art historical investigations, such as archival research, allied with dendrochronology, the documentation and study of panel maker’s punch and the Antwerp guild brand marks on the reverse of the panels. While multidisciplinary studies that investigated individual paintings (e.g. Lucas Cranach the Elder^23^ or Henri Bles^24^) or selected panel paintings by individual artists such as Rubens^18,25^ or Jordaens^26^ have been conducted before, the Jordaens Van Dyck Panel Paintings Project (JVDPPP) systematically examined 294 paintings located in over 101 different locations (museums and private collections) in 14 countries (Fig. 1a). The paintings ranged from fully finished paintings to preliminary sketches (grisaille and brunaille as well as colour) and modellos for engravings. The sizes of the paintings ranged from the very small *Head of an old man wearing a hat and holding a staff* by Anthony van Dyck, 9.1 by 8.6 cm, (Philip Mould & Company, UK) (Fig. 1b) to *The Adoration of the Shepherds* by Jacques Jordaens, 126.3 by 166.5 cm (Bristol Museum and Art Gallery, UK) (Fig. 1c).

A non- to micro-invasive method was used for the dendrochronological investigation. The measurements of the TRW of each annual ring were obtained from macro photos (ca. 5 cm segments with ca. 2 cm overlap), which were taken from the cross-section of each plank of the panels^27^. When necessary, the end grain (i.e. the edge of the panel) was cleaned with a brush or razor blade to remove dust or varnish in order to make the tree rings clearly visible. After the measurement, the TRW series were compared to a set of over 130 reference chronologies, available to the first author, and subsequently compared to other databases such as the KIK-IRPA database with over 2500 chronologies. These reference chronologies represent tree-growth patterns typical for a location or region and its environmental condition. For this study, we further combined the available reference chronologies into six larger regions (see “Methods”). The source region of each plank can thus be located based on statistical values from Student’s *t-test* (with adaptation by Baillie-Pilcher (TBP) and Hollstein (THO)^28,29^), correlations, and degree of synchronicity (so-called Gleichläufigkeit (GLK)) and visually verified. Besides this so-called dendroprovenancing, it is possible to identify the TRW series from planks that were taken from the same tree owing to almost identical growth patterns (i.e. year-to-year variability). Thus, the highest correlations are found between those planks. In addition to a high visual agreement, statistical thresholds can be applied to determine if the samples indeed originated from the same tree. Regarding oak (*Quercus* spp.), a *t*-value above ten between the TRW series likely indicates the same tree origin^30^. For TRW series with a short overlap, e.g. below 70 years, *t*-values above seven, in addition to a high visual synchronicity of the year-to-year variability, might be sufficient for an attribution of the planks to the same oak tree^31,32^. The identification of samples that originated from the same tree helps to determine which panels, and likely paintings, were produced around the same time or within a short period (within 20 years). The attribution to an artist or the date of the production of the painting cannot, however, be determined by dendrochronology alone. Only a multi-disciplinary approach can provide further insights to do so.

## Results and discussion

### The paintings

**Figure 2:**
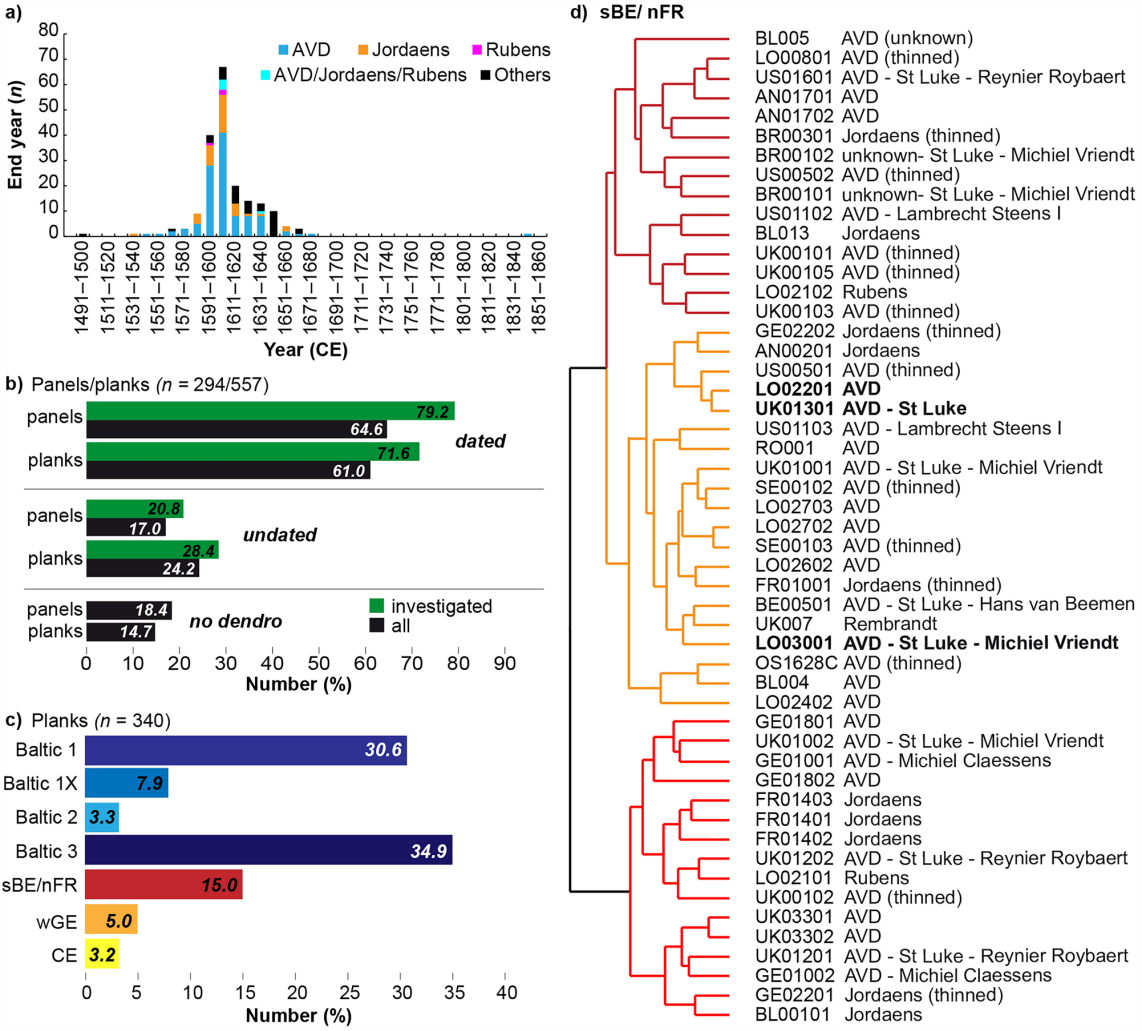
(**a**) Temporal distribution of the number of end dates for planks for paintings of individual artists (see legend for attributed artist Anthony van Dyck (AVD), Jacques Jordaens (Jordaens), Peter Paul Rubens (Rubens) and unknown). Distribution of (**b**) dated, undated, and not analysed (no dendro) panels and planks (see legend for colours) and (**c**) the main source regions of the dated planks (different Baltic groups, south-eastern Belgium/north-eastern France (sBE/nFR), western Germany (wGE) and other regions within Central Europe (CE). (**d**) Clusterogram showing TRW series (dendro code) allocated to the group south-eastern Belgium/ north-eastern France (sBE/nFR) with potential sub-groups (orange, light and dark red colours) based on Pearson correlation coefficients. Provided are related artists Anthony van Dyck (AVD), Jordaens, Rubens, or unknown artist, the presence of the Antwerp brand mark of the Guild of Saint Luke (St Luke), the name of the panel maker or if the panel was thinned). For three planks (highlighted in bold) the TRW series are shown in Fig. 3.

Overall, we investigated 157 panel paintings related to Anthony van Dyck and his workshop, a further 70 by his copyists (i.e. 17 related to Remigius van Leemput, 13 related to Theodore Russell, 40 late copies by T. S. White), 44 panel paintings related to Jacques Jordaens, eight panel paintings by Rubens, and 15 paintings by other artists or unknown Flemish masters (Fig. 2a).

From the 294 panel paintings, 190 panels could be dated (64.6%), 50 panels remained undated (17%) and for 54 panels (18.4%) dendrochronological analyses were not conducted (Fig. 2b). Regarding the latter, there were different reasons as to why it was not possible. They included an unstable state of preservation, severe thinning of the panel during previous conservation treatments, inaccessibility of the planks owing to the framing technique or it was found that a species of unmeasurable wood had been used as the painting’s support (e.g. poplar, lime or tropical wood). In terms of the 240 paintings investigated by dendrochronology, the success rate in achieving a dating was 79.2% (Fig. 2b). This involved the dating of at least one plank of the panel. Overall, a total of 126 paintings (22.6%) consisted of one plank and 108 paintings (19.4%) of two planks, while 32 paintings (5.7%) were made of three, and 18 paintings (3.2%) of four planks. The eight remaining paintings were either made of five (six paintings), seven or 10 planks (each one painting). Not all planks (82 planks, 14.7%) could be subjected to a dendrochronological analysis (Fig. 2b). From the remaining 475 measured planks, the majority (71.6%) could be dated and only 28.4% remained undated.

### Source region of the material

Ultimately, 340 planks could be dendrochronologically dated and the source region could be identified. The majority of the planks were derived from oaks from the Poland/Baltic region. This accounted for 71% of the examined planks (Fig. 2c). The exact origin within the Poland/Baltic region remains unknown since most of the Baltic tree-ring references are developed from panel paintings, and thus exported timber^33,34^. During the seventeenth century, long, straight-grained Baltic oak timber, usually converted to planks, boards, wainscots etc., was destined for export, whereas other tree species, especially pine, were used predominantly for building constructions within the region. It has to be noted that construction timber from historical buildings is the most important source for the development of local and regional tree-ring chronologies which in turn are used as reference chronologies^35^. However, Daly and Tyers^34^ were able to update the three previous Baltic reference chronology versions, so-called Baltic 1, Baltic 2 and Baltic 3^33^, and even developed two sub-groups for the Baltic 1, i.e. subgroup Baltic 1X and 1B, indicating a different sub-regional origin of the used oaks. These newly updated and classified Baltic reference chronologies can be geographically traced to coastal Lithuania (group Baltic 1) and two subregions within the Gdansk, Klaipeda and Riga region (Baltic 1X and Baltic 1B, respectively), (southern) Poland (group Baltic 2), and eastern Lithuania/north-western Russia (group Baltic 3)^34^. Our results showed that most TRW series of the Baltic/Poland sourced planks were allocated to the Baltic 3 (34.9%), followed by the Baltic 1 (30.6%), the Baltic 1X (7.9%) and Baltic 2 (3.3%) references (Fig. 2c). Interestingly, none of our TRW series matched the reference for one of the subgroups within the Gdansk, Klaipeda and Riga region, namely Baltic 1B. This could be related to the early occurrence of this material among oak panels which were found to be restricted to the period 1450–1540^34^.

Around 20% of the dated planks were sourced from western central Europe (Fig. 2c). Attempting to distinguish between different regions, regional references representing south-eastern present-day Belgium/north-eastern France (sBE/nFR) and western Germany (wGE) were developed (see methods). After allocating the individual TRW series from the measured planks based on statistical and visual agreement to each regional reference chronology, it was found that 15% (51 planks) were taken from oaks from the sBE/nFR region and 5% (17 planks) from the wGE region. Eleven planks were found to originate from other regions within Central Europe. Based on these regional allocations, new project-related TRW chronologies for the individual regions were developed when at least five TRW series were present (Supplementary Table S1 and Supplementary Fig. S1). These new TRW chronologies, spanning from 237 years (1373–1609, JVDPPP-Baltic 2) to 395 years (1251–1645, JVDPPP-Baltic 3), will serve as a reference chronology for new TRW measurements. The oaks within these groups show a high growth coherence which is expressed in the high mean inter-series correlation with values ranging from *r* = 0.42 to 0.49; similar values are reached for living oaks, for example, in the Czech Republic for well-replicated (> 300 samples) TRW sub-chronologies^36^. These high common growth patterns within our sub-groups point to a common environmental driver and thus, reflect the common source of origin. Nevertheless, also using correlation statistics and their representation in clusters, so-called clusterograms, might help identify additional sub-regions from which the oaks were sourced (Supplementary Figs. S2–7). For instance, for the sBE/nFR region (Fig. 2d), three sub-regions can likely be differentiated based on local reference chronologies which include, the Wallonia region in Belgium and both the Lorraine region and the Meuse department in north-eastern France (Supplementary Table S2). However, further research into historical forestry in these sub-regions is needed to pinpoint the exact source location of the material and to reconstruct the timber industry and trade as discussed by Daly and Tyers^34^.

### Timber characteristics

**Figure 3:**
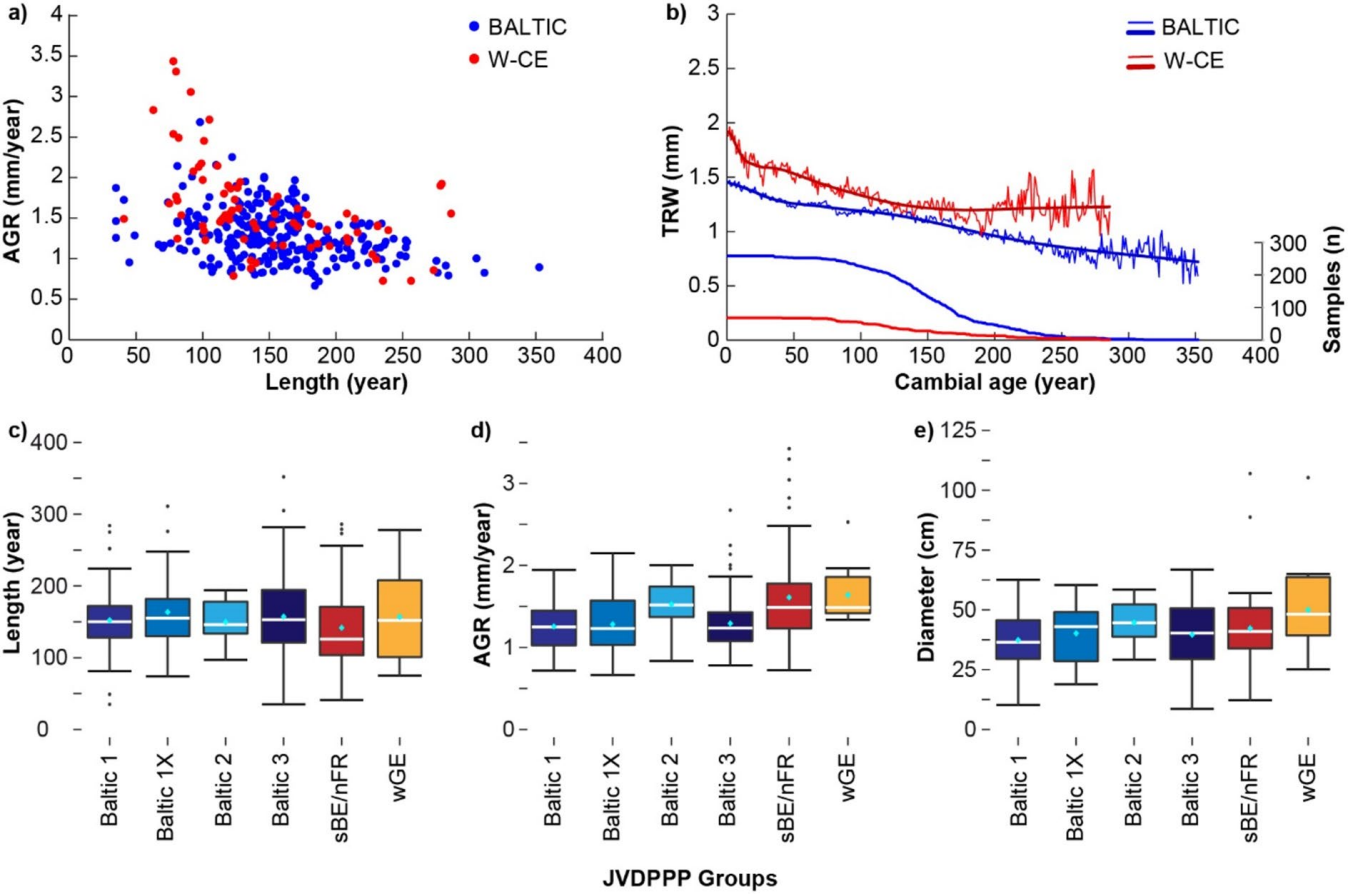
(**a**) Average growth rate (AGR, mm/year) of samples for the Baltic (blue dots) and western Central Europe (W-CE) (red dots) and (**b**) mean growth (TRW: tree-ring width) trends (upper lines) and replication (lower lines) after alignment by cambial age. Box-Whisker plots showing the (**c**) length (year), (**d**) AGR (mm/year) and (**e**) diameter (cm) for the planks allocated to the individual groups (different Baltic groups, south-eastern Belgium/north-eastern France (sBE/nFR), western Germany (wGE)).

We found that generally old and slow-grown oak trees with even, straight-grained growth were preferred for the production of the panels as stated above. Insights into this timber resource are provided by the growth rates and the number of tree rings present on the planks, and thus tree age. The average growth rate (AGR) for all Poland/Baltic material is 1.34 mm per year with a mean segment length (MSL) of 155 years and for the sBE/nFR and wGE region together (the western Central Europe group) is 1.63 mm per year for an average of 148 year-long tree-ring segments (Fig. 3a,b, Supplementary Table S2). Within the Poland/Baltic region, the planks allocated to the Baltic 2 group showed a slightly higher AGR of 1.53 mm per year at a slightly lower MSL (149 years) compared to the other Baltic groups (mean AGR of Baltic 1, Baltic 1X and Baltic 3 is 1.27 mm per year given a MSL of 157 years) (Fig. 3c,d). It has to be noted, however, that only 11 planks are included in the Baltic 2 group.

Overall, on average the oak trees used for the panels were harvested at older ages, especially when comparing with 7284 oak timbers from historical buildings and constructions in central Europe (405 BC–2008 CE), Büntgen et al.^37^ found that the statistical average is 1.56 mm per year for circa 97-year-old oak trees. Thus, these oak trees used for buildings were harvested at a younger age and showed higher annual increments. Moreover, the panels were taken from oaks of rather high diameters where the mean diameters range within the Baltic oaks from 37.4 cm (Baltic 1) to 44.7 cm (Baltic 2) and within the W-CE group, mean diameters of 42.3 cm (sBE/nFR) and 50 cm (wGE) were recorded (Fig. 3e, Supplementary Table S2). It has to be noted that the number of tree rings (length of TRW series) and tree diameters would be slightly higher given that the sapwood and the rings close to the pith were removed by the panel makers as ordered by the Guild of Saint Luke. However, it can be assumed that the majority of the oak trees used for the panels were taken from dense stands of primary forests. The tree-ring pattern did not show periodic growth reductions as seen in coppice-with-standard forest management practices^38^. This indicates that a high-forest clearing was conducted where tall mature trees were harvested from a closed canopy^39^. More archival and dendrochronological analyses are needed to obtain further insights into the existence of primary forests in the Poland/Baltic region but also western Central Europe.

### Planks from the same trees but different source regions

**Figure 4:**
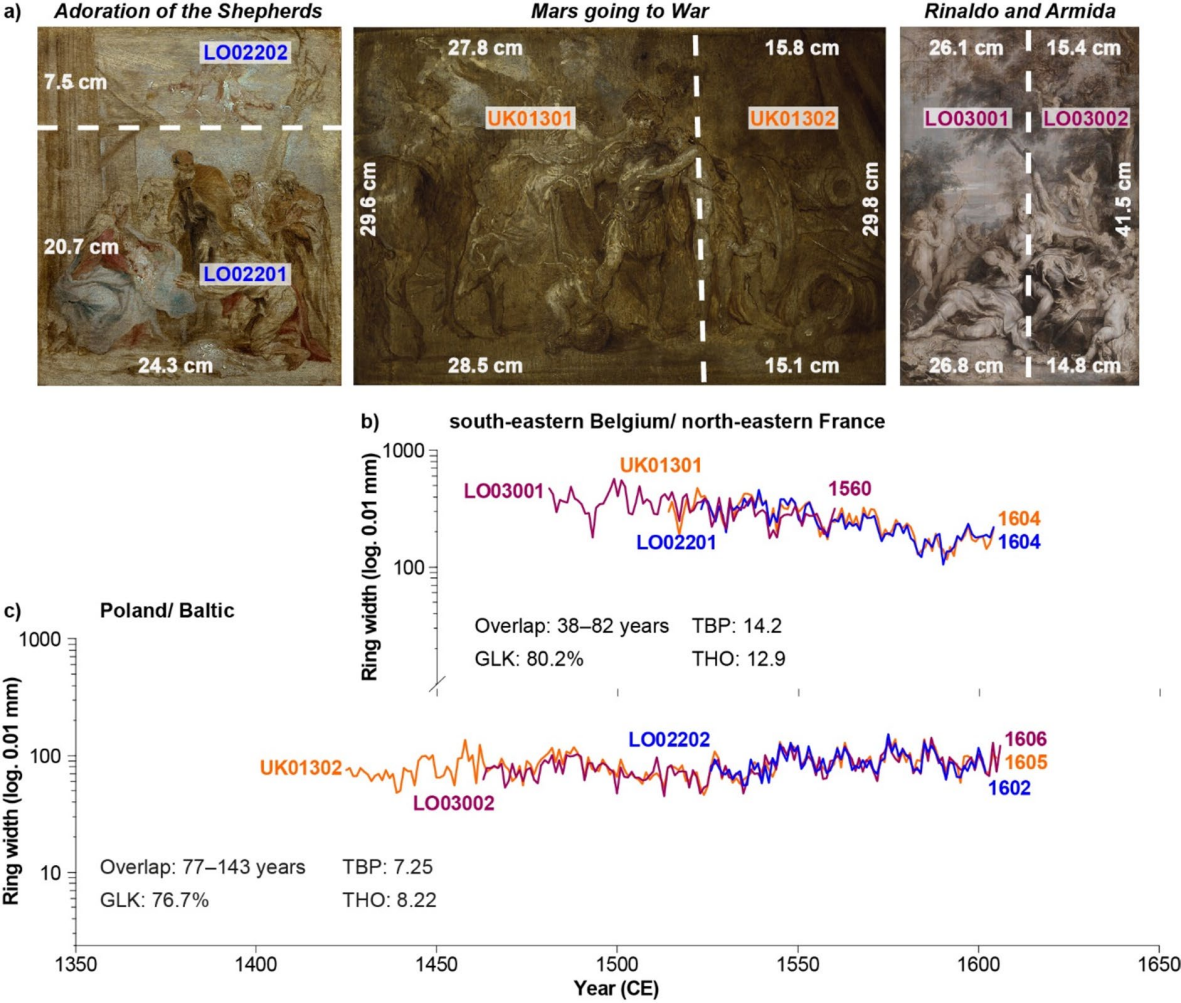
(**a**) Oil sketches of the *Adoration of the Shepherds* (left), *Mars going to War* (centre) and *Rinaldo and Armida* (right) with dimensions (white numbers) and dendro codes for each plank (colours for each painting). Raw oak tree-ring width measurements for the oak from (**b**) south-eastern Belgium/north-eastern France and (**c**) the Poland/Baltic oak in overlapping position (colours refer to planks in (**a**). Numbers are the end year of the last measured ring. Statistics show overlap (years), average synchronicity (‘Gleichläufigkeit’, GLK, in percent), the average Student’s *t*-test after Baillie and Pilcher (TBP) and Hollstein (THO).

In addition to pinpointing the source region of the used oaks, correlation statistics might help to identify planks from the same trees (Fig. 2d). For art historical questions regarding the chronological attribution of paintings from the same or different painters, the identification of planks from the same trees used for panels gained importance. Within all the dated planks, we were able to identify 89 planks from 59 paintings that matched each other (Supplementary Table S3). More specifically, planks used for the construction of the same panel support could be attributed to the same tree in 20 cases. For the planks from the remaining paintings, matches across different paintings were found and, in three cases, the planks were even taken from different source regions. These are three oil sketches related to Anthony van Dyck: *Rinaldo and Armida* (National Gallery, London), *Mars going to War* (Christ Church Picture Gallery, Oxford), and *The Adoration of the Shepherds* (The Phoebus Foundation, Antwerp) (Fig. 4). The three panels were constructed from two planks each and using dendrochronology it was possible to allocate one plank from each of the three paintings to the Poland/Baltic region and one plank to the sBE/nFR region. Here, two source regions were used for the three wooden supports. The last measured ring of the Baltic oak tree was dated to 1604 whereas the one for the oak from the sBR/nFR region was dated to 1606 indicating a similar period of tree felling. Moreover, the construction of the three panels using two planks of similar widths (only plank 2 from *Adoration of the Shepherds* is truncated) (Fig. 4a) and the use of timber from the same two trees suggest that one panel maker, in this case Michiel Vriendt (who died in 1637), produced the three panels around the same time.

Identifying planks from the same tree helps to date panels and/or planks where an older date was obtained which would likely indicate a different production time of the panel. For example, plank 1 from *Rinaldo and Armida* was dated to 1560 and only the dating of the second plank provided a younger date, i.e. a tree felling after 1606 (Fig. 4b). This gains even greater importance when the panel painting consists of one plank only. Here, different end dates of the measured planks would indicate different times of tree felling and thus, different production times, as was the case for a set of 14 portraits by Remigius van Leemput, where the planks originated from different parts of the same tree^31^.

It needs to be emphasized, however, that dating TRW series or allocating them to the same tree cannot be done by using statistics alone. Visual evaluation is always needed. Our example shows that a lower number of years in overlap (here, a minimum of 38 years) of the TRW series from the first planks of *Rinaldo and Armida*, *Mars going to War*, and *The Adoration of the Shepherds* (Fig. 4b) on their own resulted in lower correlation values (*r* = 0.51; *p* < 0. 01) and thus, would not be placed in the same group (Fig. 2d). Only visual comparison, in addition to statistics, allowed the conclusion that the three planks originated from the same tree (Fig. 4b). The TRW series for the first planks of *The Adoration of the Shepherds* and *Mars going to War*, which have a sufficient overlap of 82 years, showed higher Pearson correlation coefficients of *r* = 86 (*p* < 0.001) (Fig. 2d).

### Combining dendrochronology and archival research

**Figure 5:**
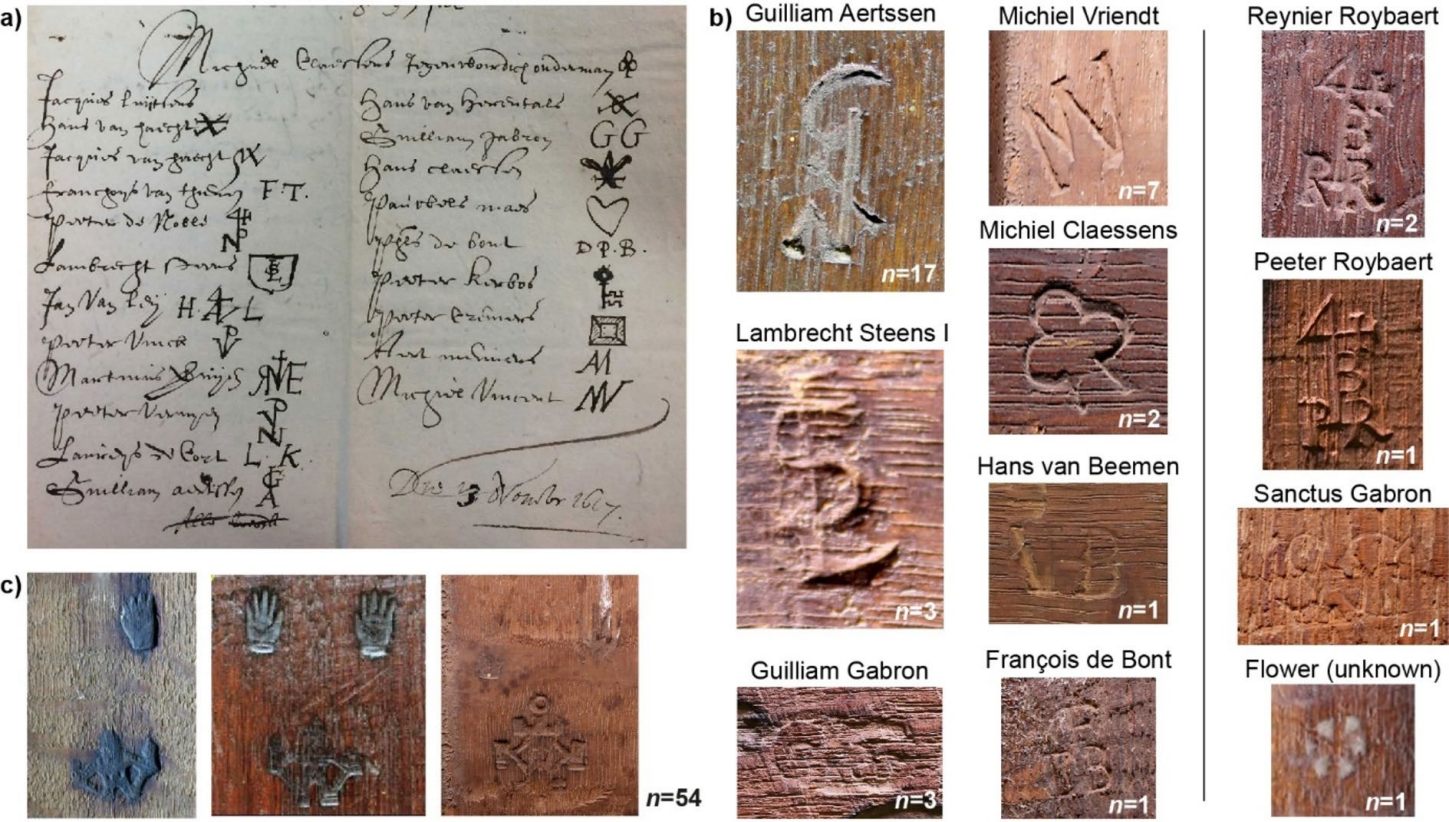
(**a**) Marks of 21 of the 22 panel makers, mirror case makers and softwood frame makers that signed the 1617 petition^15^. (**b**) Panel makers’ marks that were found on the reverse of the oak panels (left two rows) while four additional and previously unknown panel maker punch marks were identified (right row). (**c**) Different versions (from old on the left to new versions towards the right) of the quality approval mark by the Antwerp Guild of Saint Luke (ordered by ordinance of 11 December 1617^15^). Numbers (n) indicate the number of paintings with the respective mark. Raking light generates a 3D appearance of the marks.

Since the ordinance of 11 December 1617 required the removal of sapwood, that is the softer outer part of oaks which is decomposed easily by insects (especially woodworm), dendrochronology can, in most cases, only provide an earliest possible felling date (*terminus post quem* (TPQ)) of the oaks used for panel paintings^40^. Here, the active production periods of the panel makers as well as the use of the Antwerp brand mark of the Guild of Saint Luke from 11 December 1617 help to narrow down the production time of the panels if the panels are marked on the reverse^41^. Of the investigated panels, 39 panels were marked by a panel maker and 54 panels were branded with a branding iron of the Guild of Saint Luke, featuring two hands and a castle, the Antwerp coat of arms. (Fig. 5). This was important as only ten paintings contained sapwood, in which cases the estimation of the tree felling could be refined further than a simple TPQ by applying statistical estimations of the number of sapwood rings present in oak trees in Europe^4,28,40,42^.

The presence of a panel maker’s mark of Michiel Vriendt was found on the reverse of the second plank of *Rinaldo and Armida*, which was mentioned by Roy^43^ that “the initials 'MV', probably for Michiel Vriendt, [were] cut into the back of the narrower member” (Fig. 5a and b). Vriendt was active independently between 18 October 1615 when he became a free master in the Guild of Saint Luke and his death in 1637. In addition to the panel maker’s mark, the panels for *Mars going to War* and *Rinaldo and Armida* were both certified by the Guild of Saint Luke (Supplementary Fig. S8). Interestingly, one plank of *Mars going to War* displayed the two hands and a partial castle of the Antwerp brand mark of the same design as that found on *Rinaldo and Armida*, indicating that the plank had been cut from a larger panel, a theory supported by the absence of a panel maker’s mark on the panel for *Mars going to War*. The panel for the oil sketch of *Adoration of the Shepherds* was thinned and cradled during previous conservation treatment and, thus, did not reveal any marks that might once have been present. Our dendrochronological analysis revealed that the youngest dating obtained for each of the second planks was 1606 (Fig. 4c). By using the Baltic sapwood statistic, the minimum number of sapwood rings was estimated to be six rings^42^, the utilized oak for three of the planks was felled earliest after 1612 TPQ. For the drying (so-called seasoning) of the timber and transport time, an estimated minimum of two years needs to be added to this TPQ date^44,45^. Considering the presence of the marks, the panels for all three sketches were likely produced between December 1617 and 1637 by the panel maker Michiel Vriendt. A further refinement of the production of the paintings, however, can then only be provided by archival research and art historical examination. Here, the usage of different branding irons by the Antwerp Guild of Saint Luke could further provide valuable insights: the detected mark could potentially be the branding iron number four, which was in use between 1619 and 1638^46^ and references therein), however further research is needed. Overall, the case of the three Van Dycks paintings highlights that panel makers used oak planks from different geographical locations as long as they (1) were available due to an active trade network and (2) met the required quality standards by the Guild of Saint Luke. The identification of different planks from the same trees across different paintings is crucial for precise chronological attribution of the paintings and the practices of the panel makers.

To place the dendrochronological results and panel-maker marks in the correct context, art historical and archival research is essential. In the example above, the three paintings have been dated by dendrochronology and the presence of marks between the end of 1617 (potentially 1619) and 1637. Owing to their style and manner of painting, all three are confidently considered by art historians to have been painted during Van Dyck’s second Antwerp period, between 1627 and 1632, before he left for London where he died in 1641. Furthermore, the *Adoration of the Shepherds* is a preliminary sketch for the large altarpiece of the same subject that Van Dyck painted for the Onze-Lieve-Vrouwekerk in Dendermonde in 1631. Archival research revealed the significance of the panel makers Guilliam Aertssen and Michiel Vriendt (Fig. 5b) since both the young Van Dyck and Jordaens bought panels from Guilliam Aertssen in the late 1610 s/early 1620 s. However, interestingly Van Dyck changed patronage from Guilliam Aertssen in his first Antwerp period (up to 1621) to Michiel Vriendt during his second Antwerp period (1627–1632)^47^.

## Conclusion

With the dendrochronological investigation of 294 panel paintings, it was shown that oaks sourced predominantly from the Poland/Baltic region but also from the north-eastern France/south-eastern Belgium region, and western Germany, provided the wood. The first evidence of a Baltic timber trade was found in the 1970s^48^ and further advanced in later studies (e.g. Refs.^6,8,49^) with a recent update by Daly and Tyers^34^. Since the beginning of the thirteenth century, Baltic oaks were exported to Flanders, northern Belgium^6^ and since the fourteenth century to England^50^. Almost exclusively, Baltic oaks were used to make the panel supports for Flemish paintings up until the end of the sixteenth century (e.g. Ref.^13^). The different Baltic reference versions are attributed to different source regions within the larger Poland/Baltic region^34^ and even Russia^51^. From the end of the sixteenth century (mainly after 1585 with the territorial separation of Southern (Spanish) and Northern (Dutch) Netherlands) a rather radical change was observed with the appearance of the use of oak trees of western European provenance, for example, Pieter Brueghel the Younger’s small and medium-sized panels (e.g. Ref.^19^) and also by Rubens, particularly for his sketches^18^. From 142 panel paintings by different artists, Fraiture^13^ identified that as much as 29% of timber dated after 1570 CE was sourced from northeastern France (Meuse/Moselle Valley), the region around Namur and the French and Belgian Ardennes. This finding supports our results. Most of the measured outermost rings were dated between 1590 and 1640 (Fig. 2a). During this time, a political turmoil between the Southern and Northern Netherlands existed. Despite the closure of the Port of Antwerp, i.e. tolls had to be paid to the Dutch which hampered profit margins, even during the Twelve Years' Truce (9 April 1609–9 April 1621), Baltic oaks could have reached Antwerp as Dutch ships were still importing Baltic timber since the first Spanish embargo in 1585^52^. From the 1590 s onwards, the number of Dutch ships decreased as the Baltic authorities became concerned about the exploitation of their forests and other regions such as the Black Forest, Germany, agreed (first in 1593) to supply timber via the Rhine River [Ref.^52^, and references therein]. Thus, the transport of German oaks on the Moselle and Rhine rivers via Cologne, a very important Hanseatic and commercial centre, especially in the sixteenth century, was established^53^.

In general, however, the utilization of old, straight-grained, slow-grown oaks, which were regarded as high quality for panels, explains the high success rate in obtaining a dendrochronological date. The low growth rates imply the exploitation of primary oak forests, most probably the last larger ones in Europe, i.e. in the Baltic region. However, it is also remarkable that primary forests still existed in western Central Europe in the seventeenth century even though this region was densely populated and highly urbanized. Less populated and remote areas such as the Ardennes still provided straight-grained timber. Thus, tracing the origin of these mature oaks in the material of artworks requires a large data pool of regional, but also local reference chronologies, combined with further archival research on suitable forest stands and rafting history to identify the exact location of such old-grown oak forests.

## Materials and methods

### Investigation of the panel paintings

During the period 2016–2022 a systematic study of panel paintings by Jacques Jordaens (1593–1678), Anthony van Dyck (1599–1641), and their studios, as well as some collaborative works with Peter Paul Rubens (1577–1640) and copies by less known copyists such as Remigius van Leemput (1607–1675) and Theodore Russell (1614–1689) was conducted. Methods, from both the humanities and natural sciences, included art historical investigations, archival research, dendrochronology, and the study of panel maker’s punch and Antwerp guild brand marks on the reverse of the panels. Apart from the art historical and archival research, the other methods involved a physical and visual study of the panel paintings. The art historical examination and the study of the reverse of the panel were conducted using a strong light source to investigate details of the painting style and to produce raking light in order to generate better contrasts, i.e. for the panel maker marks on the reverse of a panel. For the dendrochronological investigation, the unframed panel was analysed on the end grain of the planks, which were cleaned using a toothbrush and sometimes with a razor blade to make the tree rings clearly visible. Using a camera with a macro objective mounted on a sledge with a tripod, high-resolution photos of ca. five cm sequences were taken from each plank. The ring widths were measured on these photos using CooRecorder (Cybis Elektronik & Data AB, http://www.cybis.se/forfun/dendro/) with a resolution of 0.01 mm and cross-dated using different reference chronologies and the software PAST4 (sciem.com).

### Statistical analysis

Each measured TRW series was then correlated to a set of reference chronologies and again verified against independent datasets, e.g., from the KIK-IRPA. Originally, over 130 local and regional reference chronologies are available to the authors including the five newly developed and updated Baltic references from Daly and Tyers^34^, while north-eastern France/south-eastern Belgium is composed of references B-Ardenne (Hoffsummer, ITRDB BELG001), B-Wallonia^54^, Meuse_Ardenne 2018 (update 2018 from Tegel et al.^55^), whereas western Germany is represented by Hollstein^56^ which is also composed of timber from north-eastern France, Belgium and western Germany.

Statistics verifying the best overlapping position between the individual TRW series to each other and to reference chronologies (i.e. so-called cross-dating) included the Student’s *t*-test with adaptations after Baillie and Pilcher^29^ and Hollstein^28^, the year-to-year synchronicity (‘Gleichläufigkeit’ (GLK)), and Pearson correlation coefficients (*r*) on the raw TRW data, thereby always considering the number of years of overlap.

Additionally, Pearson correlations were computed in R (version 4.0.3) using the cor() function for pairwise complete cases. Clusterograms for up to three clusters were generated for the resulting correlation matrix with the heatmaply_cor() function of the heatmaply package^57^. To calculate the tree diameter for each plank, the TRWs were cumulated and multiplied by two.

### Art historical and archival research

The art historical research followed standard academic procedures including visual examination of the front and back of the panel paintings, stylistic analysis, iconographic study, provenance research, art historical literature and connoisseurship for dating, and attribution purposes. Archival research was conducted in archives in Belgium, the Netherlands, the United Kingdom, France, Italy, Poland and Germany. Image repositories and art historians’ archives in Antwerp, the Hague, London, New York and Los Angeles were searched and consulted.

### Supplementary Information

Supplementary Information.

## Data Availability

The datasets used and/or analysed during the current study available from the corresponding author on reasonable request.
