# Supplementary material for: Timber trade in 17th-century Europe: different wood sources for artworks of Flemish painters
Source: Sci Rep. 2024 Aug 6;14:18216. doi: 10.1038/s41598-024-68641-y (PMC11303553; doi:10.1038/s41598-024-68641-y)
Supplement: Supplementary file 1 — Supplementary Information. [file 41598_2024_68641_MOESM1_ESM.docx]

**Supplementary information**

**Supplementary Table S1:** Chronology characteristics of the project (JVDPPP) chronologies for the Baltic references (32) and three regions for which more than five TRW series were allocated, namely south-eastern Belgium/ north-eastern France (sBE/nFR)) and Lower Rhine/Mosel river region (western Germany (wGE)). Provided are start and end date, the length (years), the number of included TRW series (n), the inter-series correlation (*r*), the mean segment length (MSL; years), mean sensitivity (MS), first-order autocorrelation (AR1), average growth rates (AGR; mm per year), and mean diameter (cm).

| **Code** | **Start date** | **End date** | **Length** | **n** | ***r*** | **MSL** | **MS** | **AR1** | **AGR** | **Dia** | |  |
| --- | --- | --- | --- | --- | --- | --- | --- | --- | --- | --- | --- | --- |
| JVDPPP-Baltic 1 | 1310 | 1639 | 330 | 101 | 0.49 | 152 | 0.2 | 0.58 | 1.26 | | 37.4 |  |
| JVDPPP-Baltic 1X | 1291 | 1614 | 324 | 27 | 0.47 | 163 | 0.18 | 0.59 | 1.28 | | 40.2 |  |
| JVDPPP-Baltic 2 | 1373 | 1609 | 237 | 11 | 0.42 | 149 | 0.2 | 0.58 | 1.53 | | 44.7 |  |
| JVDPPP-Baltic 3 | 1251 | 1645 | 395 | 119 | 0.46 | 157 | 0.2 | 0.62 | 1.29 | | 39.6 |  |
| JVDPPP-sBE/nFR | 1293 | 1660 | 368 | 51 | 0.48 | 141 | 0.18 | 0.67 | 1.61 | 42.3 | |  |
| JVDPPP-wGE | 1363 | 1659 | 297 | 17 | 0.47 | 156 | 0.2 | 0.61 | 1.64 | 50.0 | |  |

**Supplementary Table S2:** Cross-dating statistics for the oak mean tree-ring width series developed for the potential sub-groups 1 to 3 for the group south-eastern Belgium/north-eastern France and different independent local and regional reference chronologies. Provided are overlap (number of years), correlation of parallel run (Gleichlaeufigkeit in % (GL) (Eckstein and Bauch 1969)), significance levels (SL) (# = 95%; ## = 99%, ### = 99.9%), t-values (TBP: t-Test after transformation of the original data (raw ring-width series) using the equation after (Baillie and Pilcher 1973); THO: t-Test after transformation of the original data (raw ring-width series) using the equation after Hollstein (1980) and the dating position.

| **Sub-group** | **Reference** | **Source** | **Ol** | **GL** | **SL** | **TBP** | **THO** | **DateS** |
| --- | --- | --- | --- | --- | --- | --- | --- | --- |
| **BEFR-gr1** | **F-Lorraine** | **update 2018 Tegel et al. 2010** | **325** | **68.3** | **###** | **8.26** | **8.24** | **1617** |
| BEFR-gr1 | F-Dep. Meuse and Ardennes | update 2018 Tegel et al. 2010 | 325 | 67.1 | ### | 8.64 | 8.03 | 1617 |
| BEFR-gr1 | Lorraine Champagne und Alsace | update 2018 Tegel et al. 2010 | 325 | 67.7 | ### | 7.85 | 7.96 | 1617 |
| BEFR-gr1 | F-Dep. Meuse | update 2018 Tegel and Vanmoerkerke 2011 | 325 | 67.2 | ### | 8.27 | 7.62 | 1617 |
| BEFR-gr1 | F-Parisian Basin | Bernard 1998 | 325 | 64.6 | ### | 6.11 | 7.62 | 1617 |
| BEFR-gr1 | B-Wallonia | W. Tegel (Lab. DendroNet), unpubl. | 325 | 71.4 | ### | 7.64 | 7.53 | 1617 |
| BEFR-gr1 | France-Belgium | W. Tegel, unpubl. (DFG project) | 325 | 67.8 | ### | 7.1 | 7.49 | 1617 |
| BEFR-gr1 | B-Wallonia | W. Tegel (Lab. DendroNet), unpubl. | 325 | 66.9 | ### | 7.31 | 7.47 | 1617 |
| BEFR-gr1 | F-Dep. Vosges | update 2018 Tegel et al. 2010 | 325 | 68.6 | ### | 6.92 | 7.25 | 1617 |
| BEFR-gr1 | B-Ardennes | Hoffsummer, belg002 ITRDB | 322 | 68 | ### | 6.34 | 7.19 | 1617 |
| **BEFR-gr2** | **B-Wallonia** | **W. Tegel (Lab. DendroNet), unpubl.** | **306** | **73.7** | **###** | **11.3** | **11.9** | **1610** |
| BEFR-gr2 | F-Dep. Ardennes | update 2018 Tegel et al. 2010 | 290 | 72.8 | ### | 9.75 | 9.34 | 1610 |
| BEFR-gr2 | France-Belgium | W. Tegel, unpubl. (DFG project) | 306 | 70.1 | ### | 9.57 | 9.23 | 1610 |
| BEFR-gr2 | B-Meuse | Hoffsummer, belg003 ITRDB | 306 | 68.8 | ### | 8.59 | 9.23 | 1610 |
| BEFR-gr2 | Netherlands | mean neth001, neth015, neth016, neth017, neth018 (ITRBDB) | 306 | 67.3 | ### | 9.73 | 8.98 | 1610 |
| BEFR-gr2 | B-Wallonia | W. Tegel (Lab. DendroNet), unpubl. | 306 | 66.8 | ### | 8.43 | 8.97 | 1610 |
| BEFR-gr2 | Lorraine Champagne und Alsace | update 2018 Tegel et al. 2010 | 306 | 69.6 | ### | 9.63 | 8.75 | 1610 |
| BEFR-gr2 | F-Lorraine | update 2018 Tegel et al. 2010 | 306 | 69.9 | ### | 9.43 | 8.7 | 1610 |
| BEFR-gr2 | F-Dep. Meuse and Ardennes | update 2018 Tegel et al. 2010 | 306 | 68.6 | ### | 9.69 | 8.33 | 1610 |
| BEFR-gr2 | Germany Baden-Württemberg | W. Tegel, unpubl. (DFG project) | 306 | 65.4 | ### | 8.2 | 8.33 | 1610 |
| **BEFR-gr3** | **France-Belgium** | **W. Tegel, unpubl. (DFG project)** | **298** | **74** | **###** | **10.3** | **10.9** | **1660** |
| **BEFR-gr3** | **F-Dep. Meuse** | **update 2018 Tegel and Vanmoerkerke 2011** | **298** | **70.1** | **###** | **10.1** | **10.7** | **1660** |
| BEFR-gr3 | Lorraine Champagne und Alsace | update 2018 Tegel et al. 2010 | 298 | 73.3 | ### | 9.85 | 10.4 | 1660 |
| BEFR-gr3 | B-Wallonia | W. Tegel (Lab. DendroNet), unpubl. | 298 | 72.1 | ### | 9.9 | 10.1 | 1660 |
| BEFR-gr3 | F-Dep. Ardennes | update 2018 Tegel et al. 2010 | 298 | 68 | ### | 9.95 | 10.1 | 1660 |
| BEFR-gr3 | F-Lorraine | update 2018 Tegel et al. 2010 | 298 | 71 | ### | 9.49 | 9.97 | 1660 |
| BEFR-gr3 | B-Meuse | Hoffsummer, belg003 ITRDB | 298 | 69.8 | ### | 10.1 | 9.96 | 1660 |
| BEFR-gr3 | F-Dep. Meuse and Ardennes | update 2018 Tegel et al. 2010 | 298 | 68.6 | ### | 9.4 | 9.66 | 1660 |
| BEFR-gr3 | B-Wallonia | W. Tegel (Lab. DendroNet), unpubl. | 298 | 70.8 | ### | 9.78 | 9.63 | 1660 |
| BEFR-gr3 | F-Dep. Moselle | update 2018 Tegel et al. 2010 | 298 | 69.1 | ### | 8.35 | 9.28 | 1660 |

**Supplementary Table S3:** List of planks from panel that were allocated to another plank either from the same panel or from another panel. Provided are the information about the painting (related artist, title, location and dendro report number), the number (n) of planks used for the panel, same tree with information to the painting, the presence of the panel maker mark and the mark from the Guild of Saint Luke.

| **Painting** | **Planks (n)** | **Same tree** | **Panel maker mark** | **Guild of Saint Luke** |
| --- | --- | --- | --- | --- |
| after Anthony Van Dyck, Jan Caspar Gevaerts, Rijksmuseum, Amsterdam, NL (AM003/2017) | 1 | Anthony van Dyck, Gaspar Gevartius (1593-1666), private collection (UK014/2019) | - |  |
| Jacques Jordaens, Holy Family, St. Gilles City Hall, Brussels, Belgium (BL008/2020) | 2 | plank 1 and 2 this painting, plank 2 from Anthony van Dyck, Thomas, private collection, UK (LO00302) plank 1 from Anthony van Dyck, Paul, private collection, UK (LO01401) plank 1 and plank 2 from Anthony van Dyck, James the Greater, private collection, Germany (NL00401 and NL00402) | - |  |
| Peter Paul Rubens or Anthony Van Dyck, Jacqueline de Castres, Royal Museums of Fine Arts of Belgium, Brussels (BR006/2020) | 3 | plank 1: plank 2 from Peter Paul Rubens or Anthony Van Dyck, Jean Charles de Cordres, Royal Museums of Fine Arts of Belgium, Brussels (BR007/2020) | - |  |
| Peter Paul Rubens or Anthony Van Dyck, Jean Charles de Cordres , Royal Museums of Fine Arts of Belgium, Brussels (BR007/2020) | 3 | plank 2: plank 1 from Peter Paul Rubens or Anthony Van Dyck, Jacqueline de Castres, Royal Museums of Fine Arts of Belgium, Brussels (BR006/2020) | - |  |
| Studio of Rubens (prev. rel. to Jacques Jordaens), Head Studies of Three Women, private collection, Belgium (BR008/2020) | 2 | plank 1: plank 4 from Anthony Van Dyck, Portrait of a Man, UK (OS1545/2021) | - |  |
| Anthony Van Dyck, Philip, private collection, Germany (GE001/2019) | 2 | plank 1: plank 1 from Jacques Jordaens, Peter, Ponce, Puerto Rico (PR002/2020) | - | yes |
| Jacques Jordaens, Bust of old man crossing his hands and pulling his beard, Musee de Picardie, Amiens, France (FR009/2021) | 2 | plank 1: plank 1 and 2 from Peter by Anthony Van Dyck (private collection, UK) (LO015) plank 1: plank 1 from The Musicians by Jacob Jordaens (Prado, Madrid, Spain) | - | - |
| Jacques Jordaens, Head Study of Grapheus, Musée de la Chartreuse, Douai, France (FR011/2021) | 2 | plank 1: plank 1 from Old Man with Raised Fingers, Jordaens, Musée de la Chartreuse, Duoai, France (FR012/2021) plank 2: plank 2 from Old Man with Raised Fingers, Jordaens, Musée de la Chartreuse, Duoai, France (FR012/2021) | - | - |
| Jacques Jordaens, Old Man with Raised Fingers, Musée de la Chartreuse, Douai, France (FR012/2021) | 2 | plank 1: plank 1 from Head Study of Grapheus, Jordaens, Musée de la Chartreuse, Duoai, France (FR011/2021) plank 2: plank 2 from Head Study of Grapheus, Jordaens, Musée de la Chartreuse, Duoai, France (FR011/2021) | Guilliam Aertssen (from 1612) | yes |
| Jacques Jordaens, Mercury and Argus, Musée de la Chartreuse, Douai, France (FR014/2021) | 3 | plank 1, 2 and 3 this painting | - |  |
| Anthony Van Dyck, Thomas, private collection, UK (LO003/2021) | 2 | plank 2: plank 1 and 2 from Anthony van Dyck, James the Greater, private collection, Germany (NL004/2020), plank 2: plank 1 from Anthony van Dyck, Paul, private collection, UK (LO014/2021), plank 2: plank 1 and 2 from Jacques Jordaens, Holy Family, St. Gilles City Hall, Brussels, Belgium (BL008/2020) | Guilliam Aertssen | yes |
| attributed to Anthony Van Dyck, Christ on the Cross, Courtauld Institute Gallery, London, UK (LO008/2020) | 2 | plank 1: plank 1 from Sir Anthony Van Dyck, St. Sebastian after his Ordeal, J. Paul Getty Museum, USA (US016/2019), plank 2: plank 2 from Sir Anthony Van Dyck, St. Sebastian after his Ordeal, J. Paul Getty Museum, USA (US016/2019) | - |  |
| Studio (?) of Anthony Van Dyck, Paul, private collection, UK (LO014/2021) | 2 | plank 1: plank 1 and plank 2 from Anthony van Dyck, James the Greater, private collection, Germany (NL004/2020), plank 1: plank 2 from Anthony van Dyck, Thomas, private collection, UK (LO003/2021), plank 1: plank 1 and plank 2 from Jacques Jordaens, Holy Family, St. Gilles City Hall, Brussels, Belgium (BL008/2020) | - |  |
| Anthony Van Dyck, Peter, private collection, UK (LO015/2017) | 2 | plank 1 and 2 this painting,  planks 1 and 2: plank 1 from Jacques Jordaens, Three Musicians, Madrid, Spain (contribution by Maite Jover de Celis) | - |  |
| Studio of Rubens, Head study of a woman, Philip Mould & Company, London, UK (LO019/2017) | 3 | plank 1 and 3 this painting | Guilliam Aertssen | yes |
| Anthony Van Dyck, Adoration of the Shepherds (sketch), Philip Mould & Company, London, UK (LO022/2019) | 2 | plank 1: plank 1 from related to Anthony Van Dyck, Mars going to War, Church Gallery, Oxford, UK (UK013/2018), plank 1: plank 1 from Anthony Van Dyck, Rinaldo and Armida, National Gallery, London, UK (LO030/2019);  plank 2: plank 2 from related to Anthony Van Dyck, Mars going to War, Church Gallery, Oxford, UK (UK013/2018), plank 2: plank 2 from Anthony Van Dyck, Rinaldo and Armida, National Gallery, London, UK (LO030/2019) | - |  |
| Studio of Rubens (prev. rel. to Van Dyck), Two Head Studies of Monks, private collection, UK (LO027/2018) | 2 | 2. and plank 2 from Van Dyck, Priest in a Cope (Konstmuseum, Gothenburg, Sweden) (SE00102) 3. plank 3 from Van Dyck, Priest in a Cope (Konstmuseum, Gothenburg, Sweden) (SE00103) | - |  |
| Anthony Van Dyck, Rinaldo and Armida, National Gallery, London, UK (LO030/2019) | 2 | plank 1: plank 1 from Anthony Van Dyck, Mars going to War, Church Gallery, Oxford, UK (UK013/2018), plank 1: plank 1 from related to Anthony Van Dyck, Adoration of the Shepherds, private, UK (LO022/2019) plank 2: plank 2 from Anthony Van Dyck, Mars going to War, Church Gallery, Oxford, UK (UK013/2018), plank 2: plank 2 from related to Anthony Van Dyck, Adoration of the Shepherds, private, UK (LO022/2019) | Michiel Vriendt | yes |
| Netherlandish School, Portrait of a Man, private collection, UK (LO034/2020) | 3 | plank 2 and 3 this painting | - |  |
| Anthony Van Dyck, James the Greater, private collection, Germany (NL004/2020) | 2 | plank 1 and 2 this painting, plank 2 from Anthony van Dyck, Thomas, private collection, UK (LO003/2021), plank 1 from Anthony van Dyck, Paul, private collection, UK (LO014/2021), plank 1 and plank 2 from Jacques Jordaens, Holy Family, St. Gilles City Hall, Brussels, Belgium (BL008/2020) | Guilliam Aertssen | yes |
| Anthony Van Dyck, Portrait of a Man, The Royal Collection Trust, UK (OS1545/2021) | 4 | plank 4: plank 1 from Jacques Jordaens, Head Studies of Three Women, private collection, The Netherlands (BR008/2020) | - |  |
| Attributed to Anthony Van Dyck, Jan van den Wouwer, National Trust, UK (OS1586/2021) | 2 | plank 1 and 2 this painting | - | yes |
| attr. Remigius van Leemput, Charles I, Henrietta Maria, Charles, Prince of Wales and Princess Elizabeth, Woburn Abbey, UK (OS1588/2021) | 1 | six portraits attributed to Remigius van Leemput: Diana Russell, Countess of Newport (1624–95) (UK016/2019), Anne Villiers, Countess of Morton (d. 1684) (UK018/2019), Elizabeth Wriothesley, Countess of Northumberland (1646–90) (UK022/2019), Henrietta Boyle, Countess of Rochester (1646–87?) (UK023/2019), Barbara Villiers, Countess of Suffolk (1622–81) (UK025/2019), Penelope Naunton, Countess of Pembroke (1620–47?) (UK026/2019) | - |  |
| after Anthony Van Dyck, King Charles I, National Trust, UK (OS1602/2021) | 3 | plank 1 and 3 this painting | - |  |
| Theodore Russell, Christiana Bruce, Countess of Devonshire, Woburn Abbey Collection, UK (OS1613/2022) | 1 | Countess of Devonshire, Remigius van Leemput (Private collection, United Kingdom) (UK030) | - |  |
| Theodore Russell, Anne Russell, Countess of Bristol, Woburn Abbey Collection, UK OS1615/2022 | 2 | plank 2: Diana Newport, Countess of Bedford (OS1620/2022), and William Russell, 5th Earl, later 1st Duke of Bedford (1613-1700) (OS1623/2022) | - |  |
| Theodore Russell, Diana Newport, Countess of Bedford, Woburn Abbey Collection, UK OS1620/2022 | 1 | Anne Russell, Countess of Bristol (1616-1697) (os1615B/2022),  William Russell, 5th Earl, later 1st Duke of Bedford (1613-1700) os1623/2022) | - |  |
| Theodore Russell, Colonel Edward Russell, Woburn Abbey Collection, UK OS1621/2022 | 2 | plank 1 and 2 this painting | - |  |
| Theodore Russell, William Russell, 5th Earl, later 1st Duke of Bedford, Woburn Abbey Collection, UK OS1623/2022 | 1 | Anne Russell, Countess of Bristol (1616-1697) (os1615B/2022),  Diana Newport, Countess of Bedford (os1620/2022) | - |  |
| Anthony van Dyck, Charity, National Gallery, UK OS1628/2022 | 5 | plank 1 and 5 this painting | - |  |
| after Anthony Van Dyck, King Charles I, National Trust, UK (OS1602/2021) | 3 | plank 1 and 3 this painting | - |  |
| Jacques Jordaens, Peter, Ponce, Puerto Rico (PR002/2020) | 2 | plank 1: plank 1 from Anthony Van Dyck, Philip, private collection Germany (GE00101) | - | yes |
| Anthony Van Dyck, Judas Thaddeus, Rotterdam, NL (RO003/2020) | 2 | plank 1 and 2 this painting | - |  |
| after Anthony Van Dyck, St. Augustine in Ecstasy, Ashmolean Museum, Oxford, UK (UK003/2018) | 2 | plank 1 and 2 this painting | François de Bont | yes |
| Anthony Van Dyck, The Martyrdom of St.George, Oxford, UK (UK012/2018) | 3 | plank 1 and 2 this painting | Peeter de Noble | yes |
| Anthony Van Dyck (?), Mars going to War, Oxford, UK (UK013/2018) | 2 | plank 1: plank 1 from Anthony Van Dyck, Rinaldo and Armida, National Gallery, London, UK (LO03001), plank 1: plank 1 from related to Anthony Van Dyck, Adoration of the Shepherds, private, UK (LO022/2019);  plank 2: plank 2 from Anthony Van Dyck, Rinaldo and Armida, National Gallery, London, UK (LO03002), plank 2: plank 2 from related to Anthony Van Dyck, Adoration of the Shepherds, private, UK (LO022/2019) | - | yes |
| after Anthony Van Dyck, Gaspar Gevartius (1593-1666), private collection, UK (UK014/2019) | 1 | after Anthony Van Dyck, Jan Caspar Gevaerts, Amsterdam, NL (AM003) | - |  |
| attr. to Remigius van Leemput, Diana Russell, Royal Collection, UK (UK016/2019) | 1 | attributed to Remigius van Leemput: Anne Villiers, Countess of Morton (d. 1684) (UK018/2019), Elizabeth Wriothesley, Countess of Northumberland (1646–90) (UK022/2019), Henrietta Boyle, Countess of Rochester (1646–87?) (UK023/2019), Barbara Villiers, Countess of Suffolk (1622–81) (UK025/2019), Penelope Naunton, Countess of Pembroke (1620–47?) (UK026/2019) Charles I, Henrietta Maria, Charles, Prince of Wales and Princess Elizabeth, Woburn Abbey, UK (OS1588/2021) | - |  |
| attr. to Remigius van Leemput, Anne Carr, Royal Collection, UK (UK017/2019) | 1 | attributed to Remigius van Leemput: Frances Stuart, Duchess of Richmond (1647–1702) (UK024/2019) | - |  |
| attr. to Remigius van Leemput, Anne Villiers, Royal Collection, UK (UK018/2019) | 1 | attributed to Remigius van Leemput: Diana Russell, Countess of Newport (1624–95) (UK016/2019), Elizabeth Wriothesley, Countess of Northumberland (1646–90) (UK022/2019), Henrietta Boyle, Countess of Rochester (1646–87?) (UK023/2019), Barbara Villiers, Countess of Suffolk (1622–81) (UK025/2019), Penelope Naunton, Countess of Pembroke (1620–47?) (UK026/2019); Charles I, Henrietta Maria, Charles, Prince of Wales and Princess Elizabeth, Woburn Abbey, UK (OS1588/2021) | - |  |
| attr. to Remigius van Leemput, Frances Stuart, Royal Collection, UK (UK019/2019) | 1 | attributed to Remigius van Leemput: Katharine Howard, Lady D’Aubigny (d. 1650) (UK021/2019) | - |  |
| attr. to Remigius van Leemput, Portrait of a Lady, Royal Collection, UK (UK020/2019) | 1 | attributed to Remigius van Leemput: Portrait of a Lady (UK032/2019) | - |  |
| attr. to Remigius van Leemput, Katharine Howard, Royal Collection, UK (UK021/2019) | 1 | attributed to Remigius van Leemput: Frances Stuart, Countess of Portland (1617–94) (UK019/2019) | - |  |
| attr. to Remigius van Leemput, Elizabeth Wriothesley, Royal Collection, UK (UK022/2019) | 1 | attributed to Remigius van Leemput: Diana Russell, Countess of Newport (1624–95) (UK016/2019), Anne Villiers, Countess of Morton (d. 1684) (UK018/2019), Henrietta Boyle, Countess of Rochester (1646–87?) (UK023/2019), Barbara Villiers, Countess of Suffolk (1622–81) (UK025/2019), Penelope Naunton, Countess of Pembroke (1620–47?) (UK026/2019); Charles I, Henrietta Maria, Charles, Prince of Wales and Princess Elizabeth, Woburn Abbey, UK (OS1588/2021) | - |  |
| attr. to Remigius van Leemput, Henrietta Boyle, Royal Collection, UK (UK023/2019) | 1 | attributed to Remigius van Leemput: Diana Russell, Countess of Newport (1624–95) (UK016/2019), Anne Villiers, Countess of Morton (d. 1684) (UK018/2019), Elizabeth Wriothesley, Countess of Northumberland (1646–90) (UK022/2019), Barbara Villiers, Countess of Suffolk (1622–81) (UK025/2019), Penelope Naunton, Countess of Pembroke (1620–47?) (UK026/2019); Charles I, Henrietta Maria, Charles, Prince of Wales and Princess Elizabeth, Woburn Abbey, UK (OS1588/2021) | - |  |
| attr. to Remigius van Leemput, Frances Stuart, Duchess of Richmond, Royal Collection, UK (UK024/2019) | 1 | attributed to Remigius van Leemput: Anne Carr, Countess of Bedford (1615–84) (UK017/2019) | - |  |
| attr. to Remigius van Leemput, Barbara Villiers, Countess of Suffolk (1622-81), Royal Collection, UK (UK025/2019) | 1 | attributed to Remigius van Leemput: Diana Russell, Countess of Newport (1624–95) (UK016/2019), Anne Villiers, Countess of Morton (d. 1684) (UK018/2019), Elizabeth Wriothesley, Countess of Northumberland (1646–90) (UK022/2019), Henrietta Boyle, Countess of Rochester (1646–87?) (UK023/2019), Penelope Naunton, Countess of Pembroke (1620–47?) (UK026/2019); Charles I, Henrietta Maria, Charles, Prince of Wales and Princess Elizabeth, Woburn Abbey, UK (OS1588/2021) | - |  |
| attr. to Remigius van Leemput, Penelope Naunton, Royal Collection, UK (UK026/2019) | 1 | attributed to Remigius van Leemput: Diana Russell, Countess of Newport (1624–95) (UK016/2019), Anne Villiers, Countess of Morton (d. 1684) (UK018/2019), Elizabeth Wriothesley, Countess of Northumberland (1646–90) (UK022/2019), Henrietta Boyle, Countess of Rochester (1646–87?) (UK023/2019), Barbara Villiers, Countess of Suffolk (1622–81) (UK025/2019); Charles I, Henrietta Maria, Charles, Prince of Wales and Princess Elizabeth, Woburn Abbey, UK (OS1588/2021) | - |  |
| attr. to Remigius van Leemput, Barbara Villiers, Countess of Cleveland, Royal Collection, UK (UK027/2019) | 1 | attributed to Remigius van Leemput: Jane Needham, Mrs. Myddleton (1645–92) (UK028/2019) | - |  |
| attr. to Remigius van Leemput, Jane Needham, Royal Collection, UK (UK028/2019) | 1 | attributed to Remigius van Leemput: Barbara Villiers, Duchess of Cleveland (1641–1709) (UK027/2019) | - |  |
| attr. to Remigius van Leemput, Portrait of a Lady, Royal Collection, UK (UK032/2019) | 1 | attributed to Remigius van Leemput: Portrait of a Lady (UK020/2019) | - |  |
| Anthony Van Dyck, Adoration of the Shepherds, private collection, UK (UK033/2020) | 2 | plank 1 and 2 this painting | - |  |
| Jacques Jordaens, Portrait of a Young Married Couple, Boston, MFA, USA (US008/2019) | 4 | plank 1 and 3 this painting | - |  |
| Jacques Jordaens, Head of a Girl, Worcester Art Gallery, USA (US009/2019) | 2 | plank 1 and 2 this painting | - | yes |
| attr. To Anthony Van Dyck, St. Sebastian after Ordeal, Getty, LA, USA (US016/2019) | 2 | plank 1: plank 1, related to Sir Anthony Van Dyck, Christ on the Cross, London, UK (LO008/2020) plank 2: plank 2, related to Sir Anthony Van Dyck, Christ on the Cross, London, UK (LO008/2020) | - | yes |
| Studio of Anthony Van Dyck (?), Paul, Valenciennes, France (VA001/2019) | 2 | plank 1 and 2 this painting, planks 1 and 2 from Anthony Van Dyck, Matthew, Valenciennes, France (VA002/2019) | - |  |
| Studio of Anthony Van Dyck (?), Matthew, Valenciennes, France (VA002/2019) | 2 | plank 1 and 2 this painting, planks 1 and 2 from Anthony Van Dyck, Paul, Valenciennes, France (VA001/2019) | - |  |
| Jacques Jordaens, The Battle of the Lapiths and Centaurs, private collection (32 : 2016) | 3 | plank 1 and 2 this painting | - |  |
| Attributed to Anthony Van Dyck, Priest in a Cope, Gothenburg Museum of Art, Sweden (SE001) | 3 | plank 2: plank 2 from Studio of Rubens, Two Head Studies of Monks, private collection, UK plank 3: plank 3 from Studio of Rubens, Two Head Studies of Monks, private collection, UK |  |  |

**Supplementary figures**


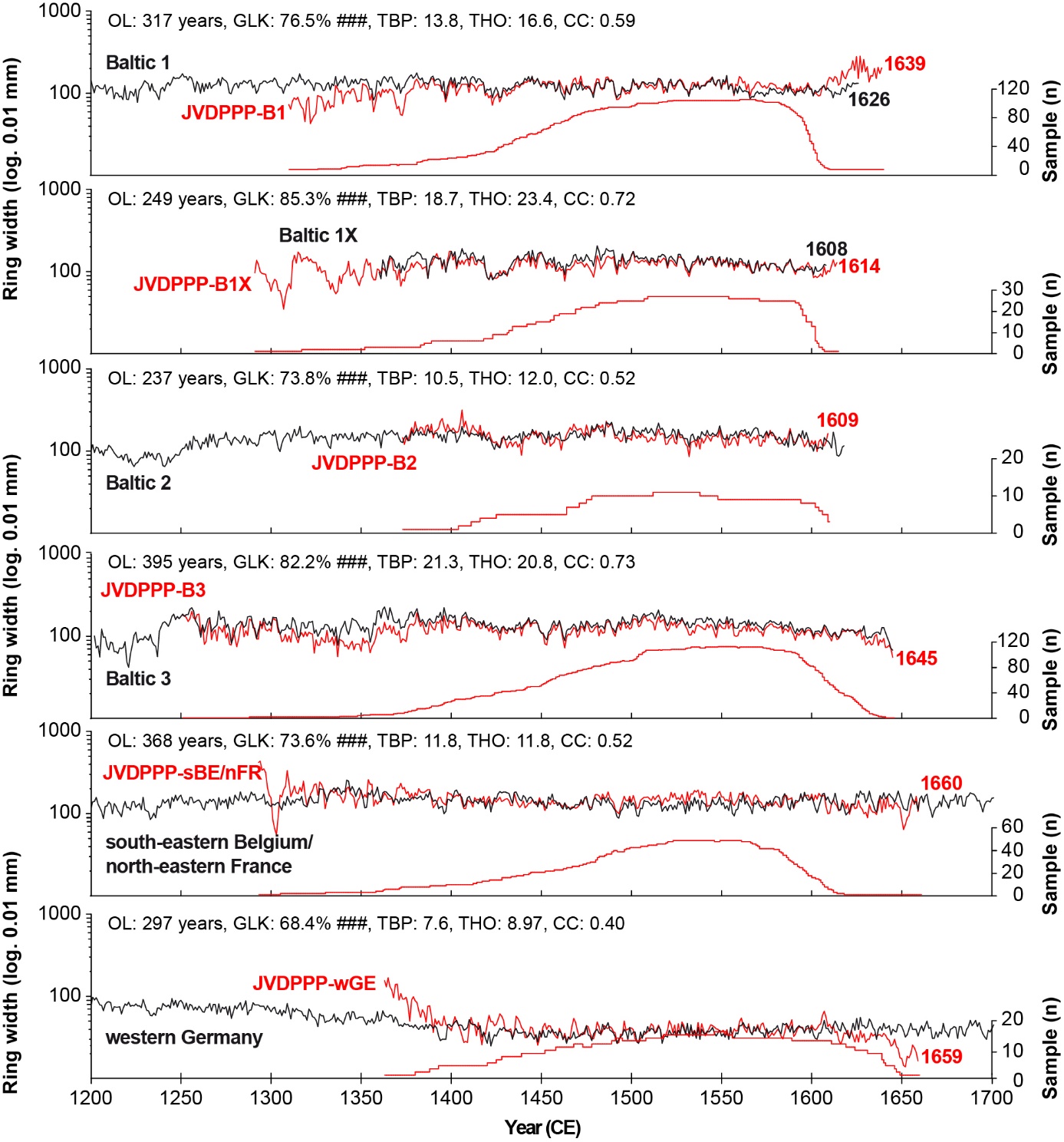


**Supplementary Figure S1:** Raw ring-width measurements for the individual JVDPPP chronologies in overlapping position with the independent reference chronologies (the Baltic references are from Daly and Tyers 2022, the remaining are developed on the individual references, see Supplementary Table 1) and the statistical evaluation of their agreement including the overlap (OL), degree of synchronicity (‘Gleichläufigkeit’ (GLK)) and significance levels (p < 0.001 (###); p < 0.05 (##)), Student’s t-test after Baillie and Pilcher (TBP) and after Hollstein (THO) and correlation coefficient (CC).


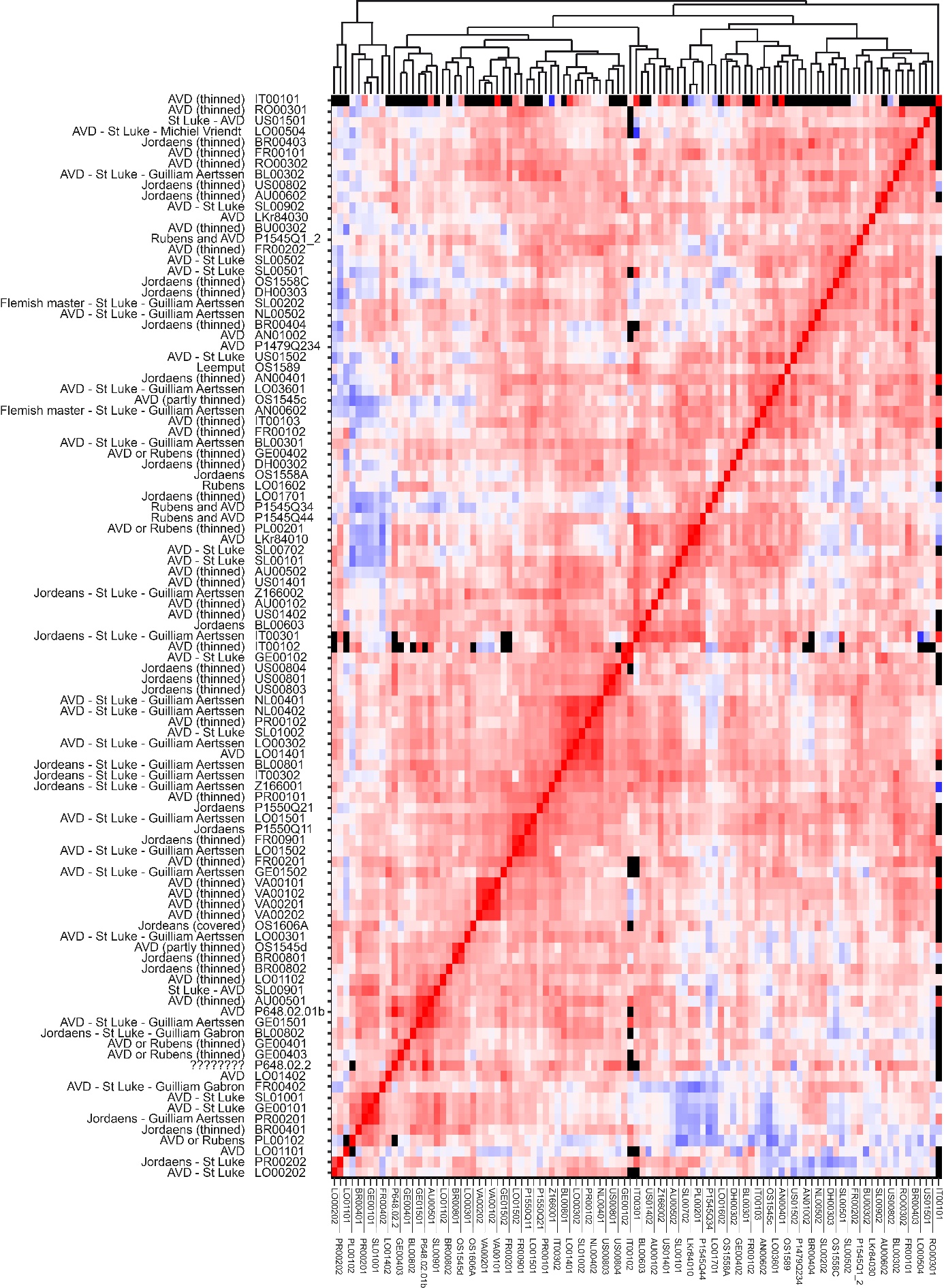


**Supplementary Figure S2:** Clusterogram for TRW series for group Baltic 1. Pearson correlations were computed in R (version 4.0.3) using the cor() function for pairwise complete cases. Clusterograms for up to three clusters were generated for the resulting correlation matrix with the heatmaply_cor() function of the heatmaply package (Moreland 2009).


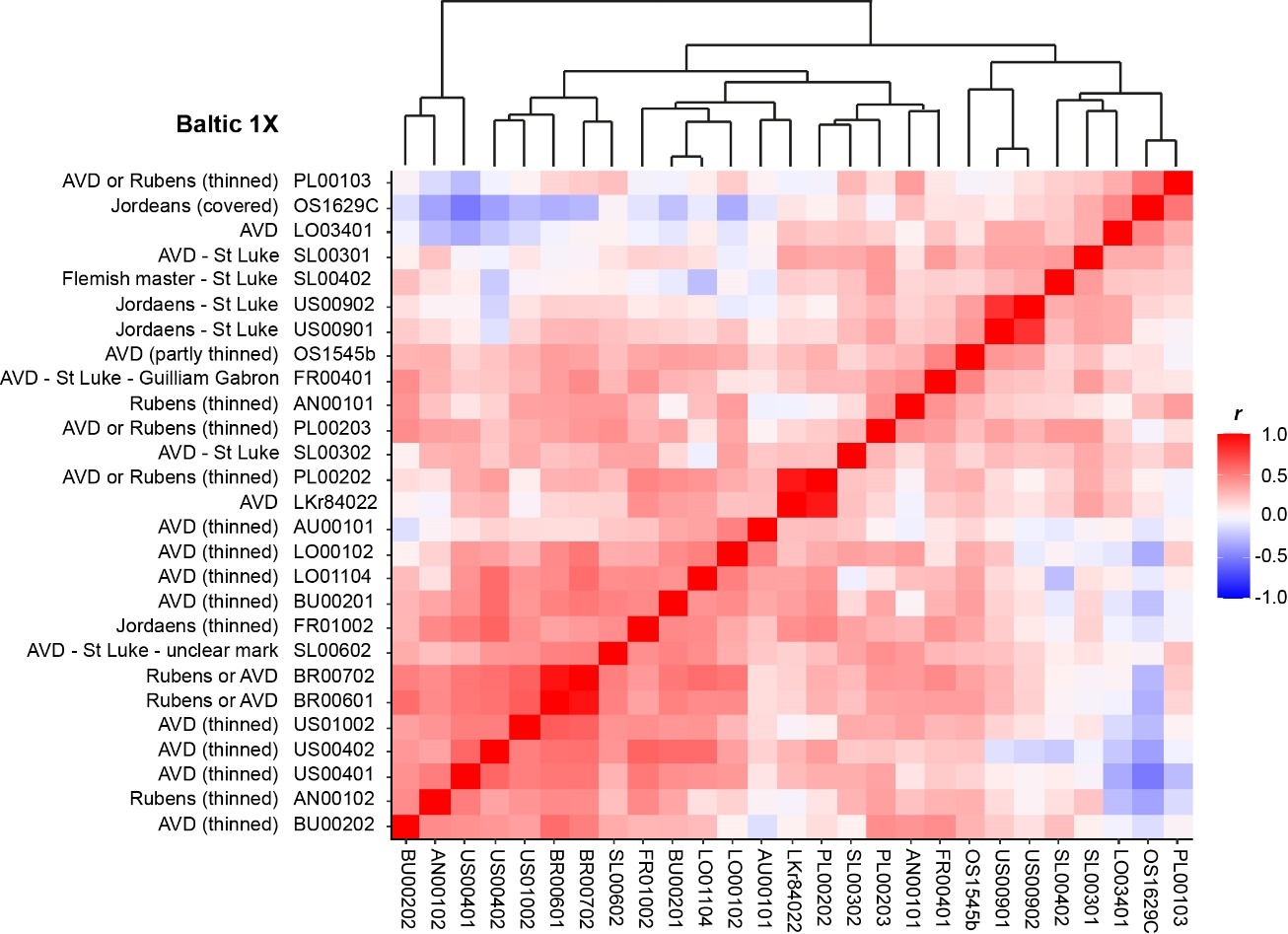


**Supplementary Figure S3:** Clusterogram for TRW series for group Baltic 1X. Pearson correlations were computed in R (version 4.0.3) using the cor() function for pairwise complete cases. Clusterograms for up to three clusters were generated for the resulting correlation matrix with the heatmaply_cor() function of the heatmaply package (Moreland 2009).


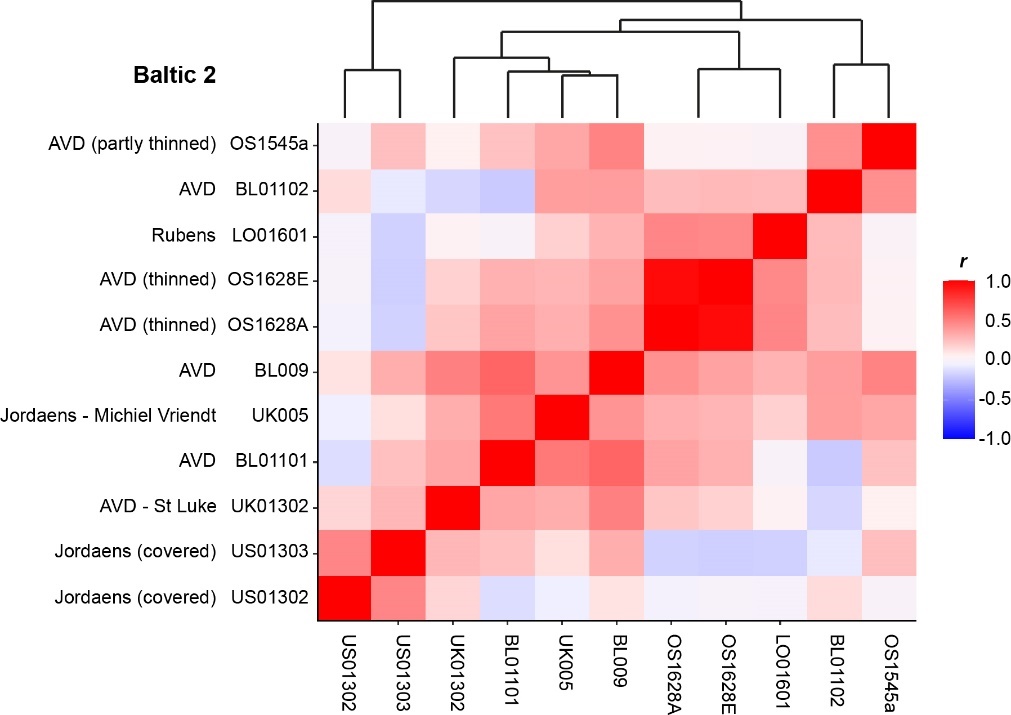


**Supplementary Figure S4:** Clusterogram for TRW series for group Baltic 2. Pearson correlations were computed in R (version 4.0.3) using the cor() function for pairwise complete cases. Clusterograms for up to three clusters were generated for the resulting correlation matrix with the heatmaply_cor() function of the heatmaply package (Moreland 2009).


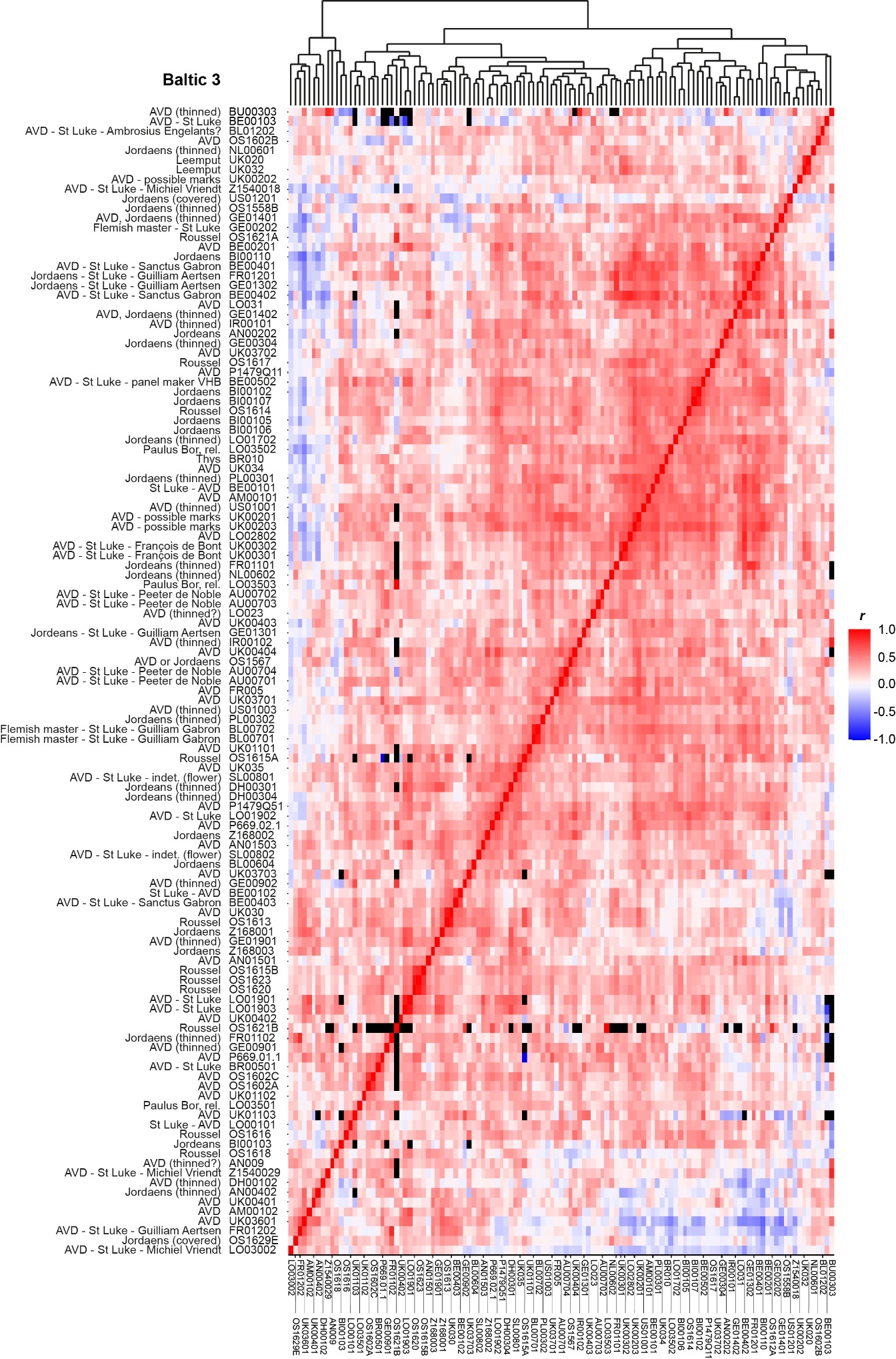


**Supplementary Figure S5:** Clusterogram for TRW series for group Baltic 3. Pearson correlations were computed in R (version 4.0.3) using the cor() function for pairwise complete cases. Clusterograms for up to three clusters were generated for the resulting correlation matrix with the heatmaply_cor() function of the heatmaply package (Moreland 2009).


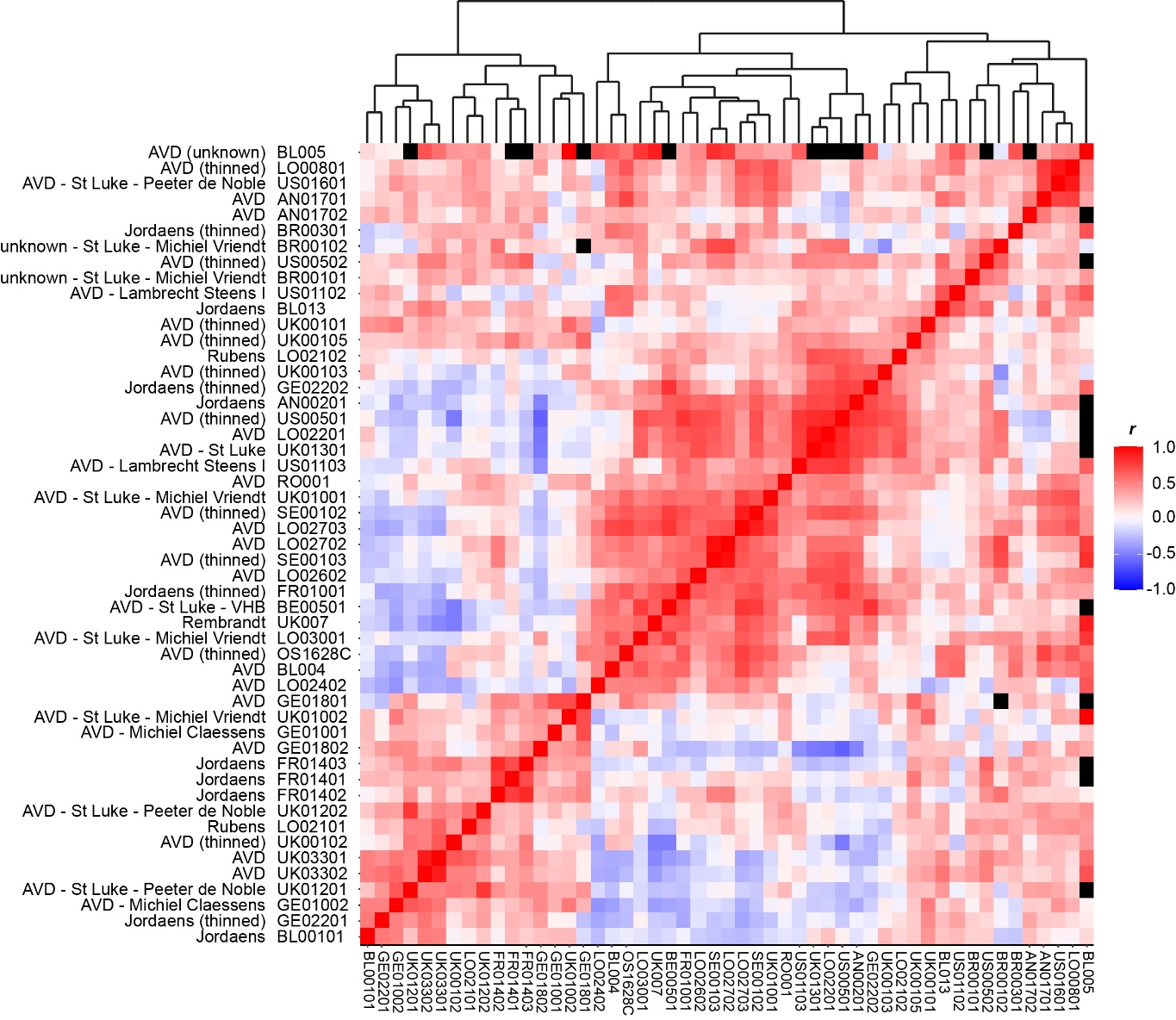


**Supplementary Figure S6:** Clusterogram for TRW series for group southeastern Belgium/ northeastern France (sBE/nFR). Pearson correlations were computed in R (version 4.0.3) using the cor() function for pairwise complete cases. Clusterograms for up to three clusters were generated for the resulting correlation matrix with the heatmaply_cor() function of the heatmaply package (Moreland 2009).


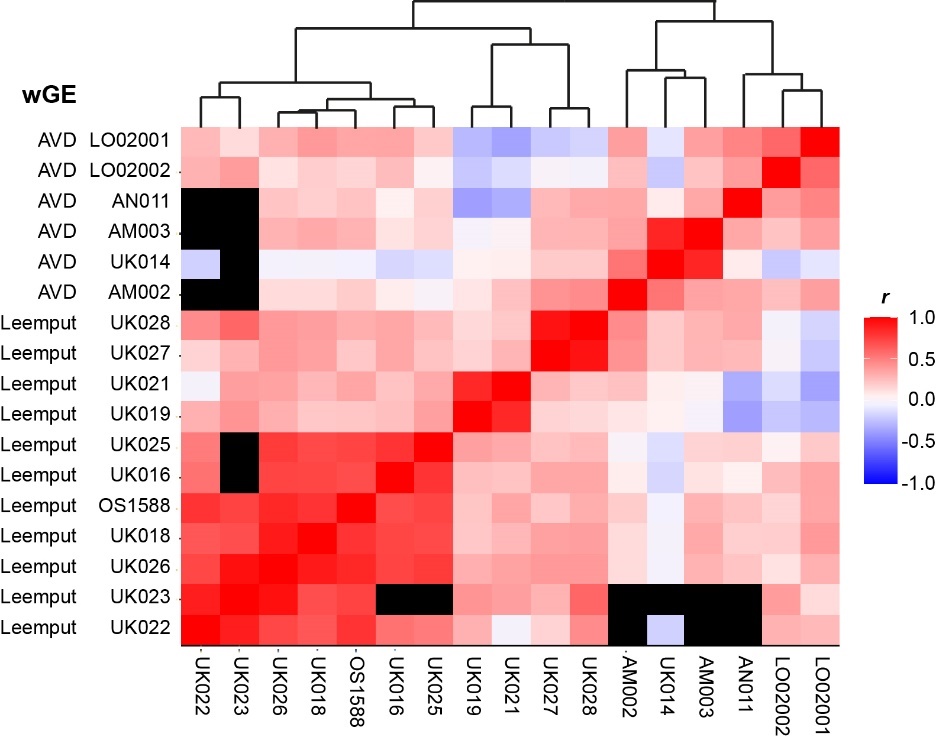


**Supplementary Figure S7:** Clusterogram for TRW series for group western Germany (wGE). Pearson correlations were computed in R (version 4.0.3) using the cor() function for pairwise complete cases. Clusterograms for up to three clusters were generated for the resulting correlation matrix with the heatmaply_cor() function of the heatmaply package (Moreland 2009).

| 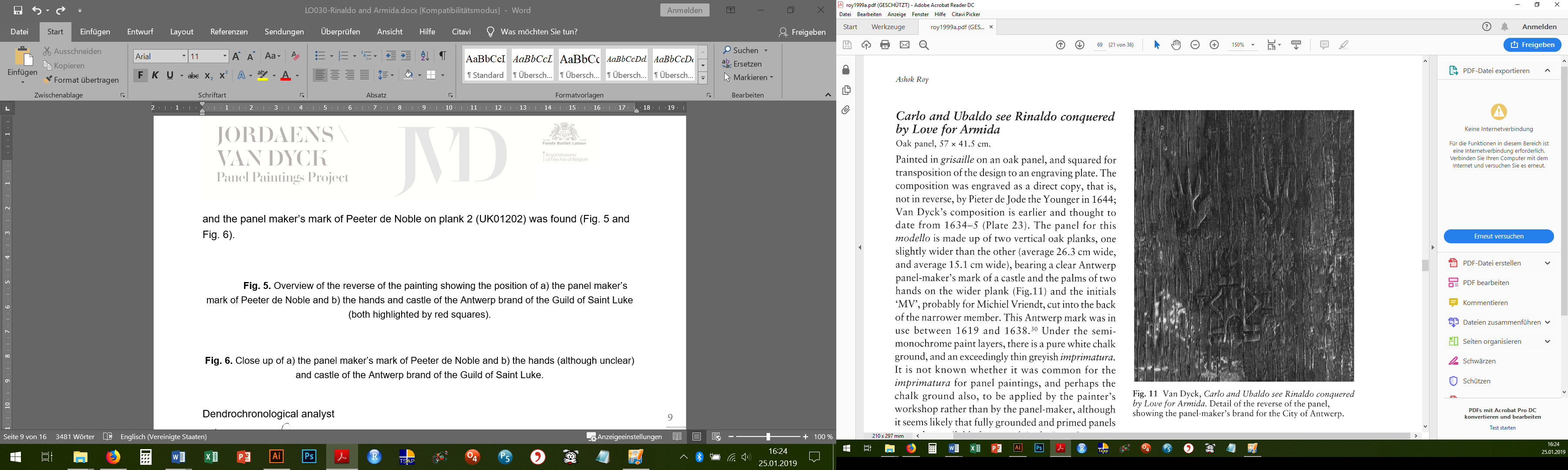 |  |
| --- | --- |

**Supplementary Figure S8:** Close-up of the Antwerp brand of the Guild of Saint Luke at the reverse of **a)** *Rinaldo and Armida*, Anthony Van Dyck (Roy 1999, p. 68) and **b)** Mars going to War, Anthony Van Dyck (Photo: A. Seim).

**References**

Baillie, Michael G. L.; Pilcher, Jonathan R. (1973): A simple crossdating program for tree-ring research. In *0041-2198*.

Bernard, Vincent (1998): L'homme, le bois et la forêt dans la France du Nord entre le Mésolithique et le Haut Moyen-Age: BAR Publishing.

Eckstein, Dieter; Bauch, Josef (1969): Beitrag zur Rationalisierung eines dendrochronologischen Verfahrens und zur Analyse seiner Aussagesicherheit. In *Forstwissenschaftliches Centralblatt* 88 (1), pp. 230–250.

Hollstein, E. (1980): Mitteleuropäische Eichenchronologie. Mainz: Philipp Von Zabern.

Moreland, K. (2009): Diverging color maps for scientific visualization. In *International symposium on visual computing*, pp. 92–103 (Springer Berlin Heidelberg).

Roy, Ashok (1999): The National gallery Van Dycks: technique and development. In *National Gallery Technical Bulletin* 20, pp. 50–83.

Tegel, Willy; Vanmoerkerke, Jan (2011): Preventive archaeology and dendrochronology: A parallel developement in Northeast France. In *Tree Rings, Art, Archaeology. Proceedings of a conference*, pp. 191–199.

Tegel, Willy; Vanmoerkerke, Jan; Büntgen, Ulf (2010): Updating historical tree-ring records for climate reconstruction. In *Quaternary Science Reviews* 29 (17-18), pp. 1957–1959.
